# Exosomal Proteomics: Unveiling Novel Insights into Lung Cancer

**DOI:** 10.14336/AD.2024.0409

**Published:** 2024-04-09

**Authors:** Guanhua Yi, Haixin Luo, Yalin Zheng, Wenjing Liu, Denian Wang, Yong Zhang

**Affiliations:** ^1^Department of Pulmonary and Critical Care Medicine and Institutes for Systems Genetics, West China Hospital, Sichuan University, Chengdu 610041, China.; ^2^Precision Medicine Center, Precision Medicine Key Laboratory of Sichuan Province, West China Hospital, Sichuan University, Chengdu 610041, China.

**Keywords:** lung cancer, exosome, mass spectrometry, proteomics

## Abstract

Although significant progress has been made in early lung cancer screening over the past decade, it remains one of the most prevalent and deadliest forms of cancer worldwide. Exosomal proteomics has emerged as a transformative field in lung cancer research, with the potential to redefine diagnostics, prognostic assessments, and therapeutic strategies through the lens of precision medicine. This review discusses recent advances in exosome-related proteomic and glycoproteomic technologies, highlighting their potential to revolutionise lung cancer treatment by addressing issues of heterogeneity, integrating multiomics data, and utilising advanced analytical methods. While these technologies show promise, there are obstacles to overcome before they can be widely implemented, such as the need for standardization, gaps in clinical application, and the importance of dynamic monitoring. Future directions should aim to overcome the challenges to fully utilize the potential of exosomal proteomics in lung cancer. This promises a new era of personalized medicine that leverages the molecular complexity of exosomes for groundbreaking advancements in detection, prognosis, and treatment.

## 1. Introduction

Globally, lung cancer remains the leading cause of cancer incidence and mortality, with an estimated 2.207 million new cases and 1.796 million deaths in 2020 alone. This represents 11.4% of all new cancer cases and 18.0% of all cancer-related deaths worldwide, underscoring the critical challenge lung cancer poses to global health [1]. Lung cancer primarily manifests as small cell lung cancer (SCLC), representing 15%, and non-small cell lung cancer (NSCLC), accounting for 80% of lung cancer cases [2]. Furthermore, NSCLC can be classified as lung adenocarcinoma (LUAD), lung squamous carcinoma (LUSC), large cell carcinoma, or bronchial carcinoid carcinoma, with LUAD being the most widespread subtype. Most SCLC patients are diagnosed with metastasis, challenging the effectiveness of current treatments such as radiotherapy and chemotherapy [3, 4]. NSCLC often goes undetected until the advanced stage, with only a 4% five-year survival rate for late-stage diagnosis compared to 83% for stage I. This stark contrast underscores the critical need for early detection in high-risk groups to improve patient outcomes [5]. The essential role of early screening and diagnosis in improving lung cancer survival cannot be overstated, especially for high-risk groups. Currently, the early detection rate is only 15%, hampered by the shortcomings of available diagnostic approaches [6]. While tissue biopsy, including methods such as fiberoptic bronchoscopy and image-guided transthoracic puncture, is pivotal for diagnosing lung cancer, these techniques are not only expensive but also fraught with potential complications and often necessitate multiple samples, imposing both physical and financial strain on patients. Conversely, low-dose spiral computed tomography (CT), despite its utility in early detection, suffers from low specificity and a heightened rate of false positives, with the added caveat of increased cancer risk due to radiation exposure [7, 8]. This landscape, however, is poised for transformation with the advent of precision medicine, promising advancements in lung cancer diagnosis and treatment.

As a pivotal component of precision medicine, liquid biopsy offers a promising avenue for the early screening of lung cancer, because of its reproducibility, minimal invasiveness, and economic viability. The focus of liquid biopsy lies in analyzing circulating tumor cells (CTCs), circulating tumor DNA (ctDNA), and exosomes [9]. There is a mounting body of evidence indicating that exosomes enriched with nucleic acids, proteins, and lipids, serve as vectors for intercellular communication, transmitting information extracellularly and playing a role in promoting the onset and progression of cancer [10-13]. Therefore, the study of exosomes and their cargoes from body fluids opens up promising avenues for lung cancer liquid biopsy. Enhanced isolation and characterization of exosomes, coupled with advancements in proteomics based on mass spectrometry (MS), now allow for detailed proteomic profiling of exosomes. Such analyses can provide critical information useful for diagnosing lung cancer early and developing individualized treatment plans [14, 15].

To delve deeper into this evolving field and gather the most recent findings, the search strategy involved retrieving English-language literature published through March 1, 2024, from databases including PubMed, Embase, and Web of Science. The following keywords were used: “Lung Neoplasms”, “Pulmonary Neoplasms”, “Neoplasms, Lung”, “Lung Neoplasm”, “Neoplasm, Lung”, “Neoplasms, Pulmonary”, “Neoplasm, Pulmonary”, “Pulmonary Neoplasm”, “Lung Cancer”, “Cancer, Lung”, “Cancers, Lung”, “Lung Cancers”, “Pulmonary Cancer”, “Cancer, Pulmonary”,“Cancers, Pulmonary”, “Pulmonary Cancers”, “Cancer of the Lung”,“Cancer of Lung”, “Exosomes”, “Exosome”, “Extracellular Vesicle”, “Vesicle, Extracellular”, “Vesicles, Extracellular”, “Exovesicles”, “Exovesicle”, “Apoptotic Bodies”, “Apoptotic Body”, “Bodies, Apoptotic”, “Body, Apoptotic”, “Proteomics”, “Peptidomics”, “Mass Spectrometry”, “Mass Spectroscopy”, “Spectral Analysis”, “Analysis, Mass Spectrum”, “Analyses, Mass Spectrum”, “Mass Spectrum Analyses”, “Spectrum Analyses, Mass”, “Mass Spectrum Analysis”, “Spectrometry, Mass”, “Spectroscopy, Mass”. The references in the reviews were manually searched to identify additional related articles. The inclusion criteria included publicly published studies exploring the link between exosomal proteomics and lung cancer, and patients who had been diagnosed with lung cancer via pathological or cytological examination. The exclusion criteria included papers not aligning with inclusion guidelines, studies missing thorough data or information, various forms of literature such as reviews, case studies, meta-analyses, conference summaries, letters, and studies that were either unavailable in full text or flagged as duplicates, and any publications found to be reproduced or duplicated. This review encapsulates the intricate journey of exosomal proteomics in lung cancer research, unveiling its promise in revolutionizing diagnostics, prognosis, and therapy amidst the challenges of heterogeneity and integration with clinical applications, and setting a path toward precision medicine.

## 2. Exosomes

Exosome generation initiated in the cell's endomembrane system through the indentation of the cell membrane, leading to early endosome formation and subsequent maturation into late endosomes [16]. These late endosomes include intraluminal vesicles (ILVs), which are shaped by the inward buckling of the endoplasmic reticulum membrane under the influence of the endoplasmic reticulum sorting complex required for transport (ESCRT) protein complex [17]. Upon ILV formation, late endosomes transform into multivesicular bodies (MVBs), which either blend with lysosomes for content breakdown or fuse with the cell membrane, releasing exosomes into the extracellular space [18].

Exosomes, versatile extracellular vesicles secreted by diverse cells, including tumor cells, are ubiquitous across various biological fluids such as blood, urine, and saliva [19]. With a phospholipid shell and a spherical or ovoid form, they house a variety of biological molecules, measuring 30-150 nm. Although the metabolic waste was first identified in 1983, subsequent research revealed its crucial role in intercellular communication, contributing to a spectrum of biological and pathological processes [13]. To further study their roles, it is necessary to isolate, purify and characterize them.

### 2.1 Isolation and characterization of exosomes

The isolation and purification processes significantly influence the integrity and composition of exosomes, which are pivotal for downstream proteomic analyses [20, 21]. A variety of methods for exosome isolation currently exist, each presenting its own set of strengths and challenges [22, 23].

Ultracentrifugation, the most widely used method for exosome isolation, involves a series of centrifugation steps to remove large particles and pellet exosomes based on their size and density [24, 25]. Although considered the gold standard, ultracentrifugation is time-consuming and labor-intensive, and may result in low purity and yield due to the co-pelleting of protein aggregates and other contaminants [26]. Density gradient centrifugation can improve the purity of isolated exosomes but requires specialized equipment and is not suitable for large-scale isolation.

Size-exclusion chromatography (SEC) separates exosomes from other components based on size, offering a simple and reproducible method that preserves the structural and functional integrity of exosomes [27]. However, SEC may co-isolate other EVs and protein aggregates of similar sizes, leading to lower purity compared to methods such as immunoaffinity capture. The purity of SEC-isolated exosomes can be improved by combining it with other techniques, such as ultrafiltration or precipitation [28].

Precipitation-based techniques, such as those employing polyethylene glycol (PEG), offer a rapid and easy way to isolate exosomes by altering their solubility [29]. Although simple and does not require specialized equipment, precipitation methods often co-precipitate non-exosomal contaminants, which can interfere with downstream analyses. Therefore, precipitation-based techniques are better suited for applications where high yield is preferred over purity.

Immunoaffinity capture, which relies on the interaction between exosomal surface proteins and their corresponding antibodies or ligands, is a highly specific method that allows for the selective isolation of exosomes [30]. This approach offers high purity and is compatible with standard laboratory equipment, making it suitable for applications requiring the analysis of specific exosome subpopulations. For instance, quantum dot-based immunoassays have been employed for the electrochemical detection of disease-specific exosomes, achieving a sensitivity of 100 exosomes/μL with high reproducibility [31]. However, the specificity and yield of immunoaffinity-based methods depend on the availability and quality of the antibodies or ligands used, and the co-isolation of non-exosomal vesicles with shared surface markers can occur.

Microfluidic-based techniques, such as acoustic nanofilters, magnetic nanowires, and the exosome total isolation chip (ExoTIC), offer rapid, efficient, and sample-sparing approaches for exosome isolation [32]. These methods combine various separation mechanisms to exploit the unique properties of exosomes and can achieve high purity and yield. However, their development and optimization can be complex, and their scalability for large-scale isolation may be challenging.

In summary, the choice of exosome isolation method depends on the specific research question, sample type, and downstream applications. Ultracentrifugation and precipitation-based methods are better suited for applications requiring high yields, while SEC and immunoaffinity capture are preferred when purity is a priority. Microfluidic-based techniques offer a balance between purity and yield but may be more complex to implement. As the field of exosome research continues to evolve, the development of standardized protocols and combinations of multiple methods will be essential for improving the purity, yield, and reproducibility of exosome isolation for various biomedical applications.

Following the isolation of exosomes, their detailed characterization becomes vital for distinguishing them from other vesicular forms. These characterization techniques can be categorized into those examining external properties, such as size and morphology, and those investigating internal composition, including specific surface markers and lipid rafts [22]. A typical three-step process is usually involved. First, microscopy techniques such as transmission electron microscopy (TEM) and scanning electron microscopy (SEM) are employed to visualize the internal and external morphology of the vesicles, respectively [33]. Second, the size distribution of the exosome population can be determined using nanoparticle tracking analysis (NTA) and dynamic light scattering (DLS) [34, 35]. By analyzing the movement of each particle, NTA can be used to calculate the hydrodynamic diameter and concentration of exosomes in the sample. This method provides a high-resolution size distribution profile and is particularly useful for detecting small changes in exosome size. DLS, also known as photon correlation spectroscopy, measures the fluctuations in scattered light intensity caused by the Brownian motion of particles in a suspension. Finally, the presence of specific surface protein markers is assessed through immunological assays such as Western blot, enzyme-linked immunosorbent assay (ELISA), and flow cytometry. These methods rely on the specific binding of antibodies to target proteins on the exosomal membrane. While there have been strides in the field of exosome isolation and characterization, exosome heterogeneity is still a problem that cannot be ignored when exosomes are used to discover biomarkers.

### 2.2 The heterogeneity of exosome and biomarker discovery

**Figure 1.**
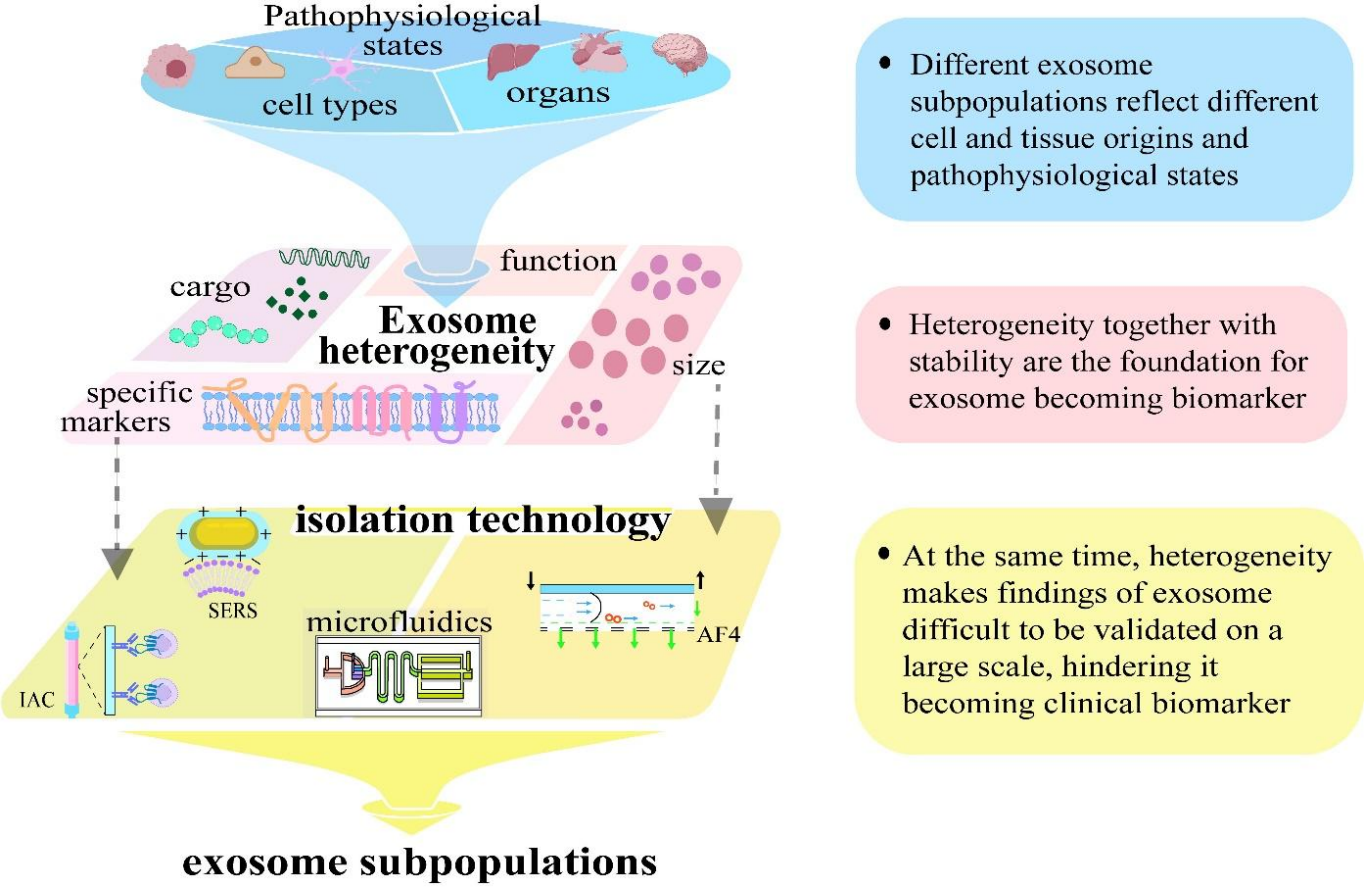
The heterogeneity of exosomes and biomarker discovery (Created with gdp.renlab.cn).

Exosome heterogeneity has emerged as a critical factor in biomarker discovery, enriching the pool of identifiable markers for a range of diseases while challenging the standards of standardization and specificity in biomarker validation. This point is concisely explained in Figure 1. This variance enables the detection of unique disease signatures through exosomal profiles, although it also complicates the process of establishing consistent, dependable biomarkers across various conditions. The diversity in exosome size, content, and functionality reflects their origin and physiological condition, influencing their development and release into the extracellular space [36].

Exosomal heterogeneity spans several dimensions, from disparities within exosome subpopulations emitted by single or similar cell types, to the assortment of exosomes derived from diverse cell origins, and their convergence with other minuscule particles [37]. Tetraspanins serve as crucial markers for delineating exosome subpopulations under specific pathological conditions [38]. Notably, pancreatic cancer and fibrosarcoma cells exhibit unique surface glycomic features, distinguishing CD9-, CD63-, and CD81-positive exosome subgroups [39, 40]. Recent findings have identified CD9-positive exosome subpopulations as progressive markers for lung cancer in HIV-positive individuals, exhibiting notably lower concentrations in the plasma of HIV-positive lung cancer patients than in that of HIV-positive individuals without cancer [41]. These insights suggest the potential utility of cancer cell-derived exosome subpopulations as liquid biopsy biomarkers for tracking cancer progression [42, 43]. Furthermore, research has highlighted the retention of cargo specific to certain pathological conditions within particular exosome subtypes [44, 45]. There is a growing interest in the relationship between unique miRNA (microRNA) clusters and EV subtypes [46, 47]. Notably, distinct miRNA profiles in Rab5b, CD9, CD31, and CD44 exosome subtypes from mouse serum have been identified, with next-generation sequencing (NGS) revealing unique miRNAs in the Rab5b and CD9 subgroups [48]. Additionally, differences in the observed exosome subpopulations across studies could be attributed to the variation in methods of isolation, conditions of storage, and techniques for characterization, despite efforts to standardize these approaches. For example, analysis of EVs from the plasma and serum of healthy subjects reveals a range of exosome subgroups, each marked by unique tetraspanin patterns. Specifically, CD9-marked exosomes are more commonly found in plasma, whereas CD63-positive exosomes are more abundant in serum. Additionally, a small subset of exosomes from blood shows markers for both CD63 and CD81, although CD81-marked EVs are rare in both fluids [49]. This finding highlights the impact of centrifuge settings and fluid type on exosome subtype selection, stressing the importance of detailed documentation in exosome research. This finding highlights the critical choice of fluid and method for exosome studies, pushing for progress in isolation techniques to improve consistency and decrease variability in findings, thereby enabling uniform and dependable exosome analysis vital for exploring their biological significance and diagnostic and therapeutic potential.

Exosomes exhibit significant diversity in terms of size, molecular signatures, and functional impacts, facilitating their organization into various subgroups [50]. With technological improvements in isolation and characterization methods, it is now possible to classify and detail exosomal subpopulations accurately [51]. This progress is essential for investigating targeted cargo loading in exosomes during different pathophysiological states [52]. Techniques currently used to isolate these subgroups focus on distinguishing features such as size and specific protein markers [53]. Gu *et al.* introduced an innovative acoustofluidic centrifugation method that utilizes acoustic forces in conjunction with fluidic vortices to swiftly separate exosomes by size in under a minute. This method effectively differentiates between large exosomes (Exo-L, 90-150 nm), small exosomes (Exo-S, 60-80 nm), and exomeres (approximately 35 nm) with high resolution [54]. Similarly, Zhang *et al.* employed AF4 to distinctively isolate exomeres (less than 50 nm), Exo-S (60-80 nm), and Exo-L (90-120 nm), revealing that the larger Exo-S and Exo-L subgroups contained differing protein compositions. Notably, Exo-L was found to have a marked inclination towards lymph nodes, highlighting its potential involvement in the metastasis of melanoma to lymph nodes [55]. Moreover, methods for isolating exosomal subgroups based on specific protein markers include immunoaffinity chromatography (IAC), microfluidics with immune-affinity, surface-enhanced Raman scattering (SERS), single-molecule array (Simoa), and aptamer techniques. The protein CD9, which is abundantly found on exosome surfaces, is critical in cancer research due to its connection with cancer development [56, 57]. Zhu *et al.* developed a high-performance liquid chromatography-immunoaffinity chromatography (CD9-HPLC-IAC) method that combines antigen-antibody interaction specificity with HPLC efficiency [58]. This approach allows for the precise isolation of CD9+ exosomes from serum, significantly minimizing protein contamination and outperforming traditional ultracentrifugation in removing serum protein interferences and size exclusion chromatography in reducing apolipoprotein contamination. The CD9-HPLC-IAC technique enhances the understanding of CD9+ exosomes in cancer mechanisms and serves as a model for isolating additional exosomal subtypes. Innovatively, the Sub-ExoProfile chip, created by Wang *et al.*, is a microfluidic platform that isolates various exosomal subgroups for detailed proteomic studies, potentially advancing cancer diagnostics and treatments [59]. Furthermore, Shen *et al.* focused on isolating EpCAM+ exosomes from lung cancer patients and demonstrated their role in cancer progression through meticulous in vivo and in vitro studies [60].

For exosomes to achieve their full potential as diagnostic and therapeutic tools, it is crucial to separate subgroups with precision. Current challenges include achieving high purity, managing variability, reducing costs, and shortening processing times. Developing or improving separation methods, with a focus on the intended use of exosomes, will facilitate better outcomes in terms of yield and purity. Identifying the cell of origin for exosomes adds another layer of complexity, exacerbated by the uncertain total number of human cell types, with estimates suggesting approximately 200 [61]. Efforts such as the Human Cell Atlas, which utilizes single-cell methodologies, have attempted to address this complexity, although they encounter obstacles such as the lack of specific markers and heterogeneity among cell states [62]. Specialized cells release EVs with distinct functionalities, but the protein markers present on exosomes do not uniquely identify their source cell or tissue [63-65]. This lack of specificity complicates the tracing of exosomes to their original cell types in various biological contexts, necessitating the development of more refined techniques and markers for accurate identification and understanding of exosomal roles in physiology and pathology. Despite the need for further refinement in exosomal subpopulation separation techniques, existing methods provide a crucial groundwork for revealing exosomal diversity and facilitating detailed subgroup analyses. Such investigations into exosomal heterogeneity are instrumental in enhancing our understanding of their involvement in diseases, pinpointing their cellular origins linked to pathological processes, and opening new avenues for diagnostics and therapeutics. Notably, research by Chanteloup *et al.* highlighted significantly elevated levels of HSP70+ exosomes in the serum of patients with metastatic breast cancer or NSCLC compared to those without metastases or healthy controls [66]. In the context of NSCLC, the presence of programmed cell death 1 ligand 1 (PD-L1) + exosomes is linked to increased cisplatin resistance, a challenge that can be overcome by blocking these exosomes [67]. Furthermore, Jin *et al.*'s next-generation sequencing analysis identified specific miRNAs in EpCAM+ exosomes as biomarkers for lung adenocarcinoma and squamous cell carcinoma [68]. In addition to cancer, extensive studies have documented the unique roles of exosomal subpopulations in diseases such as neurodegenerative disorders, liver failure, osteogenesis, and retinal conditions, underscoring the critical function of exosomes in disease pathophysiology and their promise as diagnostic and therapeutic tools [69-76].

Exosome secretion into the bloodstream and the stability of exosome components make exosomes promising non-invasive biomarkers for disease detection. However, their heterogeneity poses challenges for clinical use, primarily due to the absence of standardized methods for their study.

## 3. Advancements in proteomic technologies and analytical methods

### 3.1 Overview of proteomic technologies

Proteomics, the study of proteins composition, structure, function, and interactions within organisms, has vastly evolved from its initial methods of gel electrophoresis and immunoblotting, which were hampered by low resolution and high complexity [77]. Although two-dimensional gel electrophoresis (2D-PAGE) has facilitated advancements in resolving protein compositions and relative abundances, it is limited by its inability to separate highly acidic, basic, or hydrophobic proteins, as well as low-abundance proteins [78]. Mass spectrometry-based proteomics has emerged as a more powerful approach, further propelled by isotope labeling and high-throughput sequencing technologies [79].

Mass spectrometry has significantly propelled the quantitative analysis of proteomics forward by ionizing proteins or peptides and analyzing them based on mass-to-charge (m/z) ratios. This technique not only identifies proteins but also quantifies changes between healthy and diseased samples, which is crucial for developing classification models [80]. The bottom-up approach, which analyzes cleaved peptides, facilitates an in-depth examination of the proteome and is more suitable for larger protein sizes than the top-down method, which analyzes intact proteins and is better suited for smaller proteins [81].

Quantitative proteomics mwthods can be divided into labeled and label-free methods. Isotope labeling methods such as stable isotope labeling by amino acids in cell culture (SILAC), isobaric tag for relative and absolute quantitation (iTRAQ) and tandem mass tags (TMT) have become important tools for quantitative comparison. SILAC is an in vivo metabolic labeling method suitable for identifying changes in intracellular proteins, but it requires actively growing cells or organisms to incorporate labeled amino acids, which limits its application to certain types of clinical samples [82]. iTRAQ is an in vitro labeling method suitable for analyzing mixed samples, including clinical specimens, but it is more expensive and can suffer from issues such as ratio compression and interference from co-isolated species [83]. In contrast, TMT uses a variety of isotopically labeled reagents and is typically used to compare multiple samples under different conditions or at different time points, making it more suitable for high-throughput multiple comparison studies, but it faces similar challenges [84]. Label-free methods quantify by comparing peak areas or heights without pre-labeling, making them more suitable for complex samples and applicable to a wide range of sample types, but they are generally less accurate and reproducible than labeled methods.

In a typical "shot-gun" liquid chromatography-tandem mass spectrometry (LC-MS/MS) proteomics experiment, the main processes involved are protein extraction and digestion, mass spectrometry, data analysis and bioinformatics [85]. Data-dependent acquisition (DDA) and data-independent acquisition (DIA) are two main data acquisition methods used in mass spectrometry [86]. DIA has fewer missing values and higher sensitivity than DDA, providing more reliable data for downstream analysis, but it generates more complex data [87].

In summary, proteomic technologies have substantially advanced over the past few decades with each method offering a unique balance of advantages and limitations. Concurrent with the progress in proteomic technologies, significant developments in analytical methods and bioinformatics have also been achieved in this field.

### 3.2 Advancing analytical methods and bioinformatics in proteomics

The high-throughput data generated by mass spectrometry have spurred the innovation of numerous bioinformatics analysis methods, aimed at deciphering the complex interplay between protein regulatory mechanisms and phenotypic behaviors at the molecular level. Currently, the bioinformatics analysis workflow in bottom-up quantitative proteomics, based on mass spectrometry, typically encompasses the identification and quantification of proteins from raw data, followed by downstream bioinformatics analysis and protein network reconstruction [88]. This comprehensive approach reveals the intricate regulatory networks and their dynamic changes within biological systems, facilitating a deeper understanding of the underlying molecular processes.

The primary step of protein identification involves sequencing peptides via two strategies: database matching and de novo sequencing [89, 90]. Targeted databases containing potential protein sequences are constructed to facilitate peptide identification via peptide spectrum matching (PSM), where the accuracy of matches is crucial and depends on the selection of sophisticated search algorithms such as SEQUEST and MASCOT [91, 92]. These algorithms assess the compatibility between experimental data and theoretical models based on m/z ratios. Optimizing search parameters, such as mass tolerances, is essential for refining the identification process. Once peptides are identified, they are reconstructed into their original proteins, with longer peptides providing more reliable data for this protein inference process. The integration of both sequencing strategies enhances peptide identification accuracy. Additionally, advanced methods, such as ModifiComb, PTMselect, and Perseus, are employed to identify post-translational modifications, further detailing the complexity of the proteome and facilitating a deeper understanding of cellular functions [93-95].

Downstream analysis in proteomics research is pivotal, serving as the bridge that connects mass spectrometry-derived protein abundance data with biological insights, thereby facilitating the translation of experimental data into meaningful discoveries. This crucial phase encompasses a streamlined process of data preprocessing, which includes the essential steps of noise reduction, normalization, and imputation of missing values to enhance data quality and reliability [96]. These preparatory steps lay the groundwork for sophisticated statistical analyses, such as t-tests, analysis of variance (ANOVA), and linear models for microarray data (LIMMA), that identify significant variations in protein expression, potentially revealing disease biomarkers or key biological pathways [97, 98]. Furthermore, the application of machine learning algorithms has significantly improved the handling of large proteomics datasets [99]. By employing both supervised and unsupervised learning techniques, researchers can predict qualitative and quantitative outcomes, such as disease states or therapeutic responses, and uncover natural patterns within unlabeled datasets, facilitating novel discoveries in protein function and interaction [100, 101]. Enrichment analysis further refines the biological significance of the data by mapping identified proteins to known biological pathways, functions, or disease states, using public databases such as DAVID and STRING [102, 103]. This approach not only aids in understanding the collective role of proteins in specific biological processes but also in identifying new biomarkers or therapeutic targets. Overall, each step of downstream analysis is meticulously designed to extract precise and reliable biological interpretations from proteomic data, linking experimental findings to biological knowledge and advancing strategies for disease diagnosis and treatment in a coherent and comprehensive manner.

Protein-protein interaction (PPI) networks and signaling pathways represent pivotal areas within proteomics-based network biology, exploring complex interactions among proteins and their significant roles in disease mechanisms, notably in cancer and endocrine disorders. Affinity purification-mass spectrometry (AP-MS) serves as a principal technique for exploring PPI networks, with computational methods playing a crucial role in mitigating experimental limitations and minimizing false positives [104]. The exploration of signaling pathways has focused on elucidating the dynamic interactions between enzymes and their substrates, especially highlighting the significance of phosphorylation and other post-translational modifications (PTMs) in revealing regulatory schemes [105, 106]. These endeavors are bolstered by advanced mass spectrometry techniques and sophisticated machine learning algorithms, such as IKAP, KSEA, and KinAct, thereby providing deep biological insights that facilitate a more nuanced understanding of disease-associated signaling networks and the identification of novel therapeutic targets [107-109].

These advancements in analytical methods and bioinformatics have paved the way for a deeper understanding of the role of exosomal proteomics in various diseases. By applying these techniques to the study of exosomes, researchers can unravel the complex mechanisms underlying disease pathogenesis and progression.

## 4. The role of exosomal proteomics in various diseases

Exosomes are loaded with a diverse array of molecules such as proteins (including receptors, transcription factors, enzymes), lipids, and various forms of nucleic acids (DNA, mRNA, miRNA, and more) [110]. This composition reflects molecular profile of the originating cell, positioning exosomes as crucial in disease research [111, 112]. Notably, the protein content of exosomes offers a wealth of stable, sensitive, and distinctive insights compared to other molecular cargoes such as DNA, mRNA, and different RNA types, enhancing their utility in clinical investigations.

Exosomal proteins reside both inside the exosome and on its surface. Mass spectrometry has shown that the exosome surface harbors more than 1,000 proteins, with a high concentration of DNA- and RNA-binding proteins, including nuclear proteins such as histones [113, 114]. Surface proteins primarily consist of major histocompatibility complex (MHC) molecules, trans-membrane proteins (such as CD9, CD63, CD81, CD82, and CD53), GTPases, and membrane-associated proteins that facilitate intracellular vesicle transport and signal transduction [115-119]. Additionally, proteins such as Fas ligands, TNF receptors, transferrin receptors, integrins, and P-selectin play critical roles in interacting with target cells. Investigating exosomal surface proteins is crucial for comprehending their roles in cell communication and metastasis and advancing capture technology development. Moreover, specific surface biomarkers can differentiate between cancerous and non-cancerous exosomes, and Castillo *et al*. identified six proteins exclusive to pancreatic cancer exosomes [120].

Proteins unique to exosomes play a pivotal role in identifying and categorizing these vesicles, setting them apart from other types of extracellular vesicles, but these specific marker proteins cannot serve as the sole criteria for characterizing exosomes post-separation. Key components of the endosomal sorting complex required for transport (ESCRT), such as HGS, TSG101, VPS4, VPS32, and PDCD6IP (ALIX), act as exosomal indicators [121]. Notable exosome-specific proteins include syntenin, syndecan, MFGE8, FLOT1/2, ARF6, VAMP3, HSPA8, CD9, CD63, CD81, and CD82 [122]. These proteins not only illuminate the role of exosomes in disease development but also offer potential for disease diagnosis, prognosis, and treatment prediction. The highlighted findings, as detailed in Table 1, include the identification of PSMA7 in salivary exosomes as a biomarker for inflammatory bowel disease [123], the differential expression of proteins such as YWHAZ and BAIAP2 in plasma exosomes of patients with post-stroke cognitive impairment [124], the detection of CD133 in urinary exosomes as a marker for polycystic kidney disease [125], and the elevated levels of F2 in diabetic patients' urinary exosomes [126], showing their diagnostic potential.

**Table 1:** Advances in exosome proteomics research.

Author	Type	Source	Exosome isolation	Exosomal proteins	Function	Ref.
**Li *et al.***	Colorectal cancer	plasma	Differential centrifugation	SERPINA1, PLG	Diagnostic biomarkers	[127]
**Ding *et al.***	Gastric cancer	serum	Ultracentrifugation	Proteasome subunits (PSMA1, PSMA5, PSMB6, PSMA7, PSMA4, PSMA3, PSMB1, PSMA6)	Biomarkers and therapeutic targets for metastatic gastric cancer	[128]
**Feng *et al.***	Liver cancer	Urine	ADSP-modified arrays technology	OLFM4, HDGF, GDF15	Biomarkers of hepatocellular carcinoma	[129]
**Tomiyama *et al.***	Bladder cancer	Urine	Ultracentrifugation	HSP90, SDC1, MARCKS	Potential diagnostic biomarkers and therapeutic targets	[130]
**Iliuk *et al.***	Kidney cancer	Plasma	EVtrap (chemical affinity method)	CRKL, MTDH	Kidney cancer-specific phosphoprotein markers	[131]
**Song *et al.***	Endometrial cancer	Plasma	Exosome isolation kit	LGALS3BP	Contributed to EC growth and angiogenesis; Potential diagnostic and prognostic marker	[132]
**Zhang *et al.***	Ovarian cancer	Plasma	ExoEasy Maxi kit	GSN, FGG, FGA, LBP	Potential diagnostic biomarkers	[133]
**Wang *et al.***	Breast cancer	Serum/plasma	Precipitation solution, Ultracentrifugation	CD82	Indicators for assessing the metastatic potential of tumor cells and predicting prognosis	[134]

The role of exosomes in cancer biology is increasingly recognized, with emerging studies indicating their significant impact on tumorigenesis and cancer progression. Exosomes from donor cells can fundamentally modify the behavior of recipient cells, driving processes critical for tumor development, including transformation, proliferation, micro-environment modulation, metastasis, angiogenesis, and target organ colonization. Investigations of Wu *et al*.'s into the effects of prolonged exposure to cancer-derived exosomes revealed their potential to induce malignant transformation in urinary tract epithelial cells [135]. Further highlighting the role of exosomes in metastasis, Rodrigues *et al*. discovered that cell migration-inducing and hyaluronan-binding protein (CEMIP), which is enriched in exosomes from brain metastatic breast and lung tumors, fosters brain metastasis by creating a conducive metastatic environment [136]. Additionally, exosomal components such as extracellular matrix metalloproteinase inducers can promote tumor cell proliferation and metastasis by prompting fibroblast secretion of matrix metalloproteinases (MMPs) for extracellular matrix (ECM) remodeling [137]. Another study demonstrated that exosomal cysteine-rich receptor-like kinase (CRK) receptors secreted by bladder cancer cells enhance Erb B2/3 expression in recipient cells, facilitating vascular leakage, proliferation, and distant metastasis [138]. These findings underscore the intricate role of exosomal proteins in mediating intercellular communication within the tumor microenvironment, offering new insights into the mechanisms of cancer progression and potential therapeutic targets [139].

Exosomes secreted by tumor cells exhibit biomolecular profiles reflective of their origin, suggesting new directions for cancer biomarker discovery [140]. Research by Li *et al*. highlighted the elevation of SERPINA1 and PLG in colorectal cancer patient exosomes, surpassing traditional markers such as CEA and CA19-9 in diagnostic accuracy [127]. Furthermore, Ding *et al*.'s exploration of metastatic gastric cancer exosomes revealed the distinct expression of proteasome subunits, suggesting their role in cancer spread and serving as prospective therapeutic targets, albeit pending further elucidation of their clinical implications [128]. Innovations in exosome separation, such as the application of ADSP array technology to hepatocellular carcinoma (HCC), have streamlined the isolation of pure exosomal samples, unveiled biomarkers such as OLFM4, HDGF, and GDF15, and demonstrated the method's efficiency and potential for high-throughput diagnostic applications [129].

Additionally, the field of urologic cancer has seen advancements through exosome proteomics, with studies such as that of Tomiyama *et al*.'s identifying HSP90, SDC1, and MARCKS in bladder cancer exosomes as key to cancer pathogenesis [130]. In parallel, the EVtrap method was used to isolate the phosphoproteins CRKL and MTDH from kidney cancer exosomes, implicating them in crucial oncogenic pathways and identifying them as discriminators between cancerous and healthy states [131]. Recent advancements in exosomal proteomics have also illuminated its significance in gynecologic and breast cancer research. Ong *et al*. reported elevated LGALS3BP in endometrial cancer (EC) patient-derived plasma exosomes, suggesting that it enhances cancer cell proliferation via the PI3K/AKT/VEGFA pathway, particularly in metastatic patients [132]. Similarly, TMT-based LC-MS/MS analysis of ovarian cancer (OC) plasma exosomes identified GSN, FGG, FGA, and LBP as novel diagnostic markers, with further validation through ELISA [133]. In breast cancer studies, Wang *et al*. reported a notable reduction and tissue-to-bloodstream redistribution of the exosomal protein CD82, highlighting its diagnostic potential during cancer progression [134].

Exosomal proteomics has significantly advanced, uncovering a wealth of proteins within exosomes that play critical roles in cell communication and disease pathogenesis and as therapeutic targets. This research avenue holds promises for early disease detection and treatment, particularly in oncology and inflammatory conditions. However, challenges persist, including analytical method optimization, exosome isolation and protein quantification techniques, and the necessity for sophisticated bioinformatics to interpret complex proteomic data. In the following two sections, we will delve more deeply into the clinical potential, challenges, and future directions of exosome proteomics, focusing on lung cancer, a disease of significant concern due to its prevalence and impact on health.

## 5. Lung cancer and continuously deepening proteomics research

### 5.1 The role of proteomics in lung cancer research

Proteomics has become an essential tool in lung cancer research, offering detailed molecular insights, identifying biomarkers for early detection and prognosis, and supporting the creation of personalized treatment strategies. By leveraging efficiency and broad analytical scope of mass spectrometry, researchers can detect changes in protein expression, modifications, and interactions within lung cancer. This is achieved by examining both tumor tissues and bodily fluids, such as blood and urine [141, 142]. Despite challenges such as sample variability and noise, especially in blood-based proteomics, this approach has significantly advanced our understanding of lung cancer biology, leading to the development of innovative diagnostic and therapeutic methods [143]. Advances in lung cancer proteomics are summarized in Table 2.

**Table 2:** Advances in lung cancer proteomics.

Author	Type	Proteins	Function	Ref.
**Mao *et al.***	-	BCAT1	Promoting lung cancer metastasis	[144]
**Duan *et al.***	SCLC	ADAM12S	Promoting the proliferation, colony formation, migration, and invasion of SCLC cells	[145]
**Gasparri *et al.***	-	ARSA	Potential diagnostic markers	[146]
**Zhang *et al.***	NSCLC	FTL, MAPK1IP1L, FGB, RAB33B, RAB15	Diagnostic panel for lung cancer detection	[147]
**Böttger *et al.***	NSCLC	UGGT1, COL6A1, MAP4	Potential predictive value in determining cisplatin response to guide treatment	[148]
**Lu *et at.***	NSCLC	ARHGDIB, FN1, CDH1, KNG1	Plasma biomarker for anlotinib strati cation in NSCLC patients	[149]
**Xu *et al.***	LUAD	HSP 90β	Potential therapeutic target and prognosis biomarker	[150]
**Suwinski *et al.***	NSCLC	VEGF, OPN	Prognostic factors of radiotherapy.	[151]

One of the pivotal areas of study is the investigation of lung cancer metastasis, which is crucial for reducing mortality and enhancing patient survival rates. Significant findings, such as those from Mao *et al*., have revealed that BCAT1, a key enzyme in amino acid metabolism, is markedly elevated in metastatic lung cancer cells, and is correlated with poor survival[144]. Targeting BCAT1 to reduce its expression has been shown to inhibit metastatic spread and alter the behavior of cancer cells, implicating the modulation of factors such as SOX2 in cancer progression. Similar research on SCLC has demonstrated that ADAM12S promotes cancer cell proliferation and metastasis by affecting HK1, further emphasizing the role of proteomics in understanding cancer dynamics [145].The necessity for early detection methods is underscored by the high mortality associated with late-stage lung cancer diagnoses. Innovative proteomic analyses, such as the study by Gasparri *et al*., utilize blood samples to distinguish early-stage lung cancer patients from high-risk individuals with remarkable specificity, employing machine-learning models based on protein expression levels, notably, arysulfatase A (ARSA) [146]. Additionally, urine sample analysis has offered non-invasive diagnostic alternatives that complement traditional imaging techniques, identifying biomarkers that can differentiate lung cancer from other diseases [147].

In the context of treating advanced NSCLC, where options are limited and chemoresistance poses a significant challenge, platinum-based chemotherapy remains the standard treatment. Proteomics has opened new avenues for personalizing treatment, as evidenced by the findings of Böttger *et al*., who identified protein markers that predict cisplatin sensitivity in NSCLC cell lines [148]. This potential for tailored therapy aims to prevent patients from undergoing ineffective and harmful treatments. Furthermore, Lu *et al*.'s work on NSCLC patients treated with anrotinib identified plasma biomarkers that could predict therapeutic effectiveness, illustrating the ability of proteomics to refine treatment selection and enhance patient outcomes [149]. Moreover, the role of proteomics in improving lung cancer prognosis has been highlighted through studies such as those conducted by Xu *et al*., which linked high levels of HSP90β to poor prognosis and identified potential therapeutic targets [150]. Research by Suwinski *et al*. further confirmed the value of proteomics, showing that elevated levels of proteins such as VEGF and OPN in patient serum are significant prognostic markers, providing insights into future treatment and management strategies for lung cancer [151].

Together, these studies underscore the transformative impact of proteomics in lung cancer research.

### 5.2 Expanding insights: the emergence of glycoproteomics in lung cancer

Building on the foundation established by proteomics in lung cancer research, glycoproteomics emerged as an equally critical field, extending the depth of our investigation into lung cancer. These sugar attachments, known as glycosylations, are crucial for a variety of cellular processes, including immune responses, cell growth, and signaling [152, 153]. Altered glycosylation patterns are often associated with cancer development, aiding tumor growth and spread by affecting cell adhesion and signaling [154-157]. By focusing on the study of glycoproteins and their complex glycosylation patterns, glycoproteomics offers new insights into the mechanisms driving lung cancer progression and provides novel biomarkers and therapeutic targets.

Expanding on these insights, glycoproteomics significantly contributes to identifying different lung cancer types by analyzing unique glycosylation patterns. For instance, Hirao *et al*. employed lectin to isolate NSCLC-specific glycoproteins, identifying unique markers that distinguish between adenocarcinoma and large cell carcinoma, despite similar protein expression levels [158]. This distinction is further supported by studies revealing distinct glycosylation patterns in exosomes from different lung cancer types, suggesting that glycosylation profiles can reflect the histological features of cancer [159]. The use of lectins for biomarker discovery has proven valuable, as showen in studies where specific glycoproteins, such as PON1 and AACT, were identified as potential markers for early-stage NSCLC [160, 161]. Furthermore, research by Lu *et al*. on FUT4-linked fucosylation revealed its impact on cancer-related cellular processes [162]. Beyond biomarker discovery, glycoproteomics plays a crucial role in identifying glycosylation inhibitors, offering new avenues for cancer therapy. For example, Alvarez *et al*. discovered that the drug pictilisib alters glycosylation processes in lung cancer cells, potentially reducing their malignancy [163]. This finding aligns with findings that manipulating glycosylation can either increase sensitivity to treatments such as cisplatin or enhance resistance, highlighting the significance of glycosylation in cancer treatment [164].

The field of lung cancer glycoproteomics has significantly advanced, revealing the roles of various glycosylation patterns in the onset and progression of lung cancer. This field allows for the identification of unique glycoprotein markers, enhancing early diagnosis and monitoring. It also opens pathways for new treatments and inhibitors targeting glycoproteins, potentially controlling cancer growth and resistance. However, challenges remain, such as the need for broader validation of findings and improved techniques for glycoprotein enrichment and isolation to further investigate the roles of glycosylation in lung cancer.

### 5.3 Clinical application potential of lung cancer exosome proteomics

**Figure 2.**
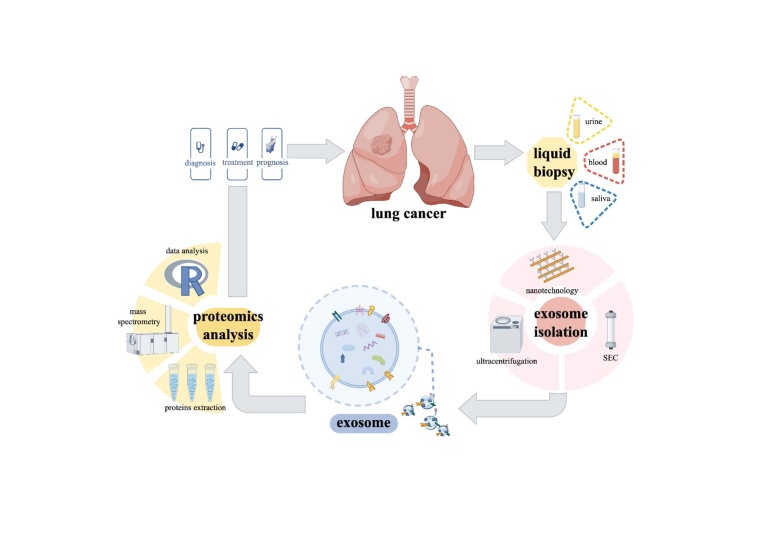
Workflow of exosome proteomics in lung cancer (By Figdraw).

Exosome proteomics has become an essential tool in lung cancer research, offering insights into the underlying mechanisms of cancer progression and pinpointing potential therapeutic targets, as well as diagnostic and prognostic biomarkers. Tumor-derived exosomes influence critical biological processes such as angiogenesis, invasion, and cell proliferation, thereby contributing to tumor growth and metastasis [165]. This has led to increased interest in using exosomes for liquid biopsy applications [166]. Significant progress has been made in isolating and analyzing exosomal proteins from body fluids such as saliva and serum, despite challenges posed by the presence of abundant proteins such as albumin, IgG, and amylase, which can obscure exosomal content [167]. The aforementioned advancements in separation techniques and mass spectrometry have significantly improved the purity, quantity, and analytical depth of exosomal proteins, facilitating the identification of more reliable biomarkers. The current workflow for lung cancer exosome proteomics is illustrated in Figure 2. Plasma-derived exosomal proteins potentially surpass traditional blood-based biomarkers, such as tumor-associated antigens (TAAs) and tumor-associated autoantibodies (TAAbs), by providing comprehensive and specific information with enhanced stability and sensitivity [168]. These findings are crucial for early lung cancer diagnosis, monitoring disease progression, and evaluating treatment response. Their ability to provide detailed insights even in complex biological samples underlines their potential in transforming lung cancer diagnostics and treatment, setting a new direction for clinical applications in oncology. Recently, identified potential biomarkers in lung cancer through exosome proteomics are summarized in Table 3.

**Table 3:** Potential biomarkers identified via lung cancer exosomal proteomics.

Author	Type	Source	Exosome isolation	Exosomal proteins	Function	Ref.
**Sandfeld-Paulsen *et al.***	-	Serum	Extracellular vesicle array	CD151, CD171, Tetraspanin 8	Separators of patients with cancer of all histological subtypes versus patients without cancer	[169]
**Jeong *et al*.**	NSCLC	Serum	A disposable 10-mL column	GCC2	Potential diagnostic biomarker	[170]
**Wang *et al*.**	NSCLC	Serum	Ultracentrifugation	LBP	Candidates of metastatic NSCLC	[171]
**Gao *et al*.**	NSCLC	Serum	Precipitation solution	Tim-3, Galectin-9	Correlated with several malignant parameters	[172]
**Liu *et al*.**	LUAD	Serum	Ultracentrifugation	ITGAM, CLU	Potential diagnostic biomarker	[173]
**Pedersen *et al*.**	SCLC	Serum	Ultracentrifugation	Coagulation factor XIII A, Complement factor H-related protein 4	Potential diagnostic biomarker	[174]
**Sun *et al*.**	-	Saliva	Ultracentrifugation	MUC5B, IQGAP	-	[175]
**Li *et al*.**	NSCLC	Urine	Ultracentrifugation	LRG1	Potential diagnostic biomarker	[176]
**Jin *et al*.**	-	Urine	Centrifugation and SEC	WASL, STK10 and WNK1	lymphocyte migration regulation related proteins, Potential diagnostic biomarker	[177]
**Luo *et al*.**	NSCLC	plasma	Ultracentrifugation	FGB, FGG and VWF CFHR5, C9 and MBL2	Potential diagnosis biomarkers, potential biomarkers for NSCLC metastasis	[178]
**Baran *et al*.**	NSCLC	serum	Exosome isolation reagent	IL-34, HLA-DMA, HLA-DOB	Potential diagnostic biomarker, related to cancer-associated fibroblasts (CAFs) and tumor-associated macrophages (TAMs) infiltration processes	[179]

A notable advancement in this field was demonstrated by Sandfeld-Paulsen *et al.*, who utilized extracellular vesicle arrays to phenotype plasma-derived exosomes from lung cancer patients and controls, and identified CD151, CD171, and tetraspanin 8 as potential early diagnostic markers[169]. By further refining the search for LUAD markers, Liu *et al*. identified ITGAM and CLU in serum exosomes through comprehensive LC-MS/MS and western blot analyses [176]. Moreover, elevated levels of Tim-3 and Galectin-9 in plasma exosomes from NSCLC patients were considered as promising candidates for NSCLC diagnosis and prognosis, highlighting their correlation with an aggressive cancer phenotype [172]. The exploration extends beyond plasma to include diverse bodily fluids. Pedersen *et al*. revealed coagulation factor XIII A and complement factor H-associated protein 4 in SCLC patients' plasma-derived microvesicles and exosomes, revealing a novel avenue for the early detection of SCLC [174]. Similarly, the analysis of salivary and urinary-derived exosomes uncovered significant protein markers such as MUC5B, IQGAP, LRG1, and SPARCL1, expanding the diagnostic landscape to incorporate non-invasive bodily fluids [175, 176, 180]. Further enriching the diagnostic toolkit, Jin *et al*.’s study on urine exosomes identified differential expression of WASL, STK10, and WNK1 as potential lung cancer biomarkers [177]. These findings, along with the identification of FGB, FGG, and VWF in exosome panels, fortify the prospect of early NSCLC diagnosis and shed light on the correlation of survival duration, emphasizing the significance of exosomal proteins in lung cancer prognosis [178]. Moreover, the identification of CFHR5, C9, and MBL2 as biomarkers for NSCLC metastasis assessment, along with interleukin-34, HLA-DMA, and HLA-DOB in serum exosomes, underscores the intricate role of exosomal proteins in NSCLC progression and immune system interactions [178, 179]. This finding is complemented by the correlation of FAM166B, Killer Cell Immunoglobulin-like Receptor 2DL1, and Olfactory Receptor 52R1 with lymph node metastasis, providing a prognostic perspective on NSCLC metastatic spread [179].

With respect to the molecular underpinnings of lung cancer, research by Jeong *et al*. and Wang *et al*. elucidated the role of specific exosomal proteins in indicating disease progression and metastatic potential, emphasizing their therapeutic relevance [170, 171]. Similarly, Feng *et al.*'s work on the role of exosomal ITGB6 in activating CAFs and ECM remodeling shed light on the pathways governing tumor dormancy and recurrence in LUAD, suggesting that targeted interventions targeting the ITGB6-KLF10-TGF-β axis are novel therapeutic strategies [181]. Han *et al.*'s insights into the altered tumor microenvironment following osimertinib resistance in NSCLC underscore the strategic potential of manipulating exosome-mediated immune modulation to counteract drug resistance [182]. Furthermore, the identification of THBS2+ CAFs as key players in promoting aggressiveness in early-stage LUAD not only highlights the role of exosomes in tumor-stroma communication but also presents a viable target for therapeutic exploitation [183].

Collectively, these findings not only enrich our understanding of the molecular landscape of lung cancer but also herald the advent of exosome-based strategies for the comprehensive management of lung cancer, revealing the promise of exosome proteomics in heralding breakthroughs in diagnosis, prognosis, and treatment. This forward momentum, however, is tempered by existing challenges within the field, necessitating a discussion on the obstacles faced and future directions for exosomal proteomics research in lung cancer.

## 6. Challenges and future directions in lung cancer exosome proteomics

### 6.1 Standardization of exosome proteomics research: ensuring reliability and reproducibility

Exosome heterogeneity, as discussed in the previous sections, poses significant challenges to the reliability of research findings in exosome proteomics. Recent advances in separating exosomal subpopulations have partially mitigated these challenges, enhancing their potential as precise biomarkers.

To ensure transparency and reproducibility in exosome research, the field has established guidelines for various stages of the research process. The Minimal Information for Studies of Extracellular Vesicles 2023 (MISEV2023) provides a comprehensive framework for the isolation, characterization, and reporting of exosomes [184]. Adherence to these guidelines is essential for maintaining the quality and comparability of research findings across different studies and laboratories. The initiative encourages the submission of protocols to EV-TRACK, promoting a more transparent and consistent research environment [185]. In addition to standardized experimental procedures, databases such as Vesiclepedia and ExoCarta contain extensive information on the molecular profiles of exosomes, serving as valuable resources for benchmarking and data interpretation [186, 187].

Moreover, the development of user-friendly data analysis platforms, such as Firmiana, has greatly enhanced the accessibility and standardization of mass spectrometry data analysis [188]. These platforms streamline the bioinformatics analysis process from raw MS data to the generation of biological insights. Furthermore, proteomics studies in the field should also comply with established reporting guidelines, such as the Minimum Information About a Proteomics Experiment (MIAPE) and other recognized standards for proteomics data interpretation [189, 190].

### 6.2 Emerging frontier proteomics technologies: paving the way for single-exosome proteomics in lung cancer

#### 6.2.1 Advancing mass spectrometry innovation: deepening exosome proteome exploration

The recently introduced Orbitrap Astral mass spectrometer represented a quantum leap in the functional analysis of proteomes, offering unparalleled speed, sensitivity, and comprehensive coverage [191]. By combining the Orbitrap analyzer with the innovative asymmetric track lossless (Astral) analyzer, this instrument enabled ultrafast label-free quantification and comprehensive proteome profiling, achieving up to 100 full yeast proteomes per day or 48 human proteomes per day with a depth of approximately 10,000 human protein groups in just half an hour [192, 193]. The Orbitrap Astral mass spectrometer also excelled in phosphoproteomics, mapping approximately 30,000 unique human phosphorylation sites within a mere half-hour of data collection [194], and confidently identifying over 16,000 phosphopeptides in a single half-hour LC-MS/MS run using minimal peptide inputs [195]. The parallelized acquisition of the Orbitrap and Astral analyzers enabled high-throughput quantitative analysis, delivering substantial improvements over previous methods [196]. Using data-independent acquisition, the instrument quantified five times more peptides per unit time than state-of-the-art Orbitrap mass spectrometers, producing high-quality quantitative measurements across a wide dynamic range [197]. Finally, the Orbitrap Astral mass spectrometer hold immense potential for single-cell proteomics, accurately defining cellular states and unraveling the complexity of human physiology at the single-cell level [198]. As the field of proteomics continues to evolve, the Orbitrap Astral mass spectrometer is poised to play a pivotal role in unlocking new insights and driving groundbreaking discoveries [199]. Advances in mass spectrometry technology have also had a profound impact on the study of exosome proteomes. We anticipate that this cutting-edge mass spectrometer will enable comprehensive exosome proteomic characterization with unprecedented depth and accuracy, ushering in a new era of exosome proteome exploration.

#### 6.2.2 Multiplatform approach for exosome proteomics

In addition to the continuous advancements and updates in mass spectrometry technology to enhance the coverage depth of exosomal proteomics, the utilization of multiplatform proteomic approaches also facilitates the achievement of this objective and augments biological insights. For instance, the complementary strengths of mass spectrometry-based label-free techniques and the high sensitivity of targeted immunoassays based on proximity extension assays (PEA) have been demonstrated in plasma proteomics [200], yet their combined advantages in the proteomics of various biofluid exosomes require further exploration and validation. Developed by Olink Proteomics in Uppsala, Sweden, PEA represents an innovative proteomic technology that combines the specificity of quantitative real-time PCR with the versatility of multiplex immunoassays. The unique feature of PEA is its mechanism of target biomarker detection, where two antibodies, each tagged with a distinct DNA oligonucleotide, bind simultaneously to their target, enabling precise and dual recognition. This technology is well-suited for the analysis of low-abundance proteins within complex biological matrices. Furthermore, the capacity of PEA to measure proteins within extracellular vesicles has been recognized [201]. Despite the higher costs associated with Olink Proteomics kits and the extended research timelines in comparison to mass spectrometry, the future promises an increasing convergence of various protein detection methods to further enrich the depth and breadth of exosomal proteomics.

#### 6.2.3 Single-cell proteomics and spatial proteomics

Advances in single-cell proteomics (SCP) and spatial proteomics have opened up new avenues for investigating cellular heterogeneity, protein dynamics, and disease mechanisms at an unprecedented level of resolution [202]. However, the connection between these cutting-edge technologies and exosomal proteomics remains to be further explored and elucidated.

SCP has emerged as a groundbreaking field that enables proteomic analysis of individual cells, offering insights into cellular heterogeneity and disease mechanisms that are often overlooked by bulk analyses. Advances in experimental design, sample preparation, separation techniques, and MS instrumentation have undoubtedly fueled the progress in SCP. However, the isolation of single cells from complex samples such as tissues or biofluids remains a critical and challenging step prior to MS analysis. Techniques such as fluorescence-activated cell sorting (FACS), magnetic-activated cell sorting (MACS), laser capture microdissection (LCM), microfluidics, and manual cell picking/ micromanipulation have been employed to isolate rare cells of interest from their surrounding milieu [203]. Nevertheless, the efficiency and accuracy of these techniques may vary, and the potential for introducing artifacts or biases during the isolation process cannot be overlooked. Several MS-based approaches, including mass cytometry, mass spectrometry imaging (MSI), and single-cell proteomics by mass spectrometry (SCoPE-MS), have been developed for SCP analysis [204, 205]. Although these techniques have shown promising results, they also have limitations. For instance, mass cytometry, although capable of analyzing multiple proteins simultaneously, is limited by the number of available metal isotopes and may not provide comprehensive coverage of the proteome [206]. Similarly, MSI, while providing spatial information, may have lower sensitivity and resolution than other MS techniques [207]. The aforementioned technical aspects of single-cell proteomics may be similarly applicable to single-exosome proteomics. However, due to the significant differences between exosomes and cells in terms of size, composition, and biophysical properties, further experimental validation is necessary to extend the scope of these technologies to the field of single-vesicle proteomics. Therefore, the adaptation of single-cell proteomics techniques to single-exosome proteomics requires careful optimization and validation to ensure the reliability and reproducibility of the results.

Spatial proteomics, an interdisciplinary field, investigates protein localization and dynamics within subcellular compartments and tissues, providing critical insights into cellular functions and disease mechanisms. At present, three principal and complementary spatial proteomics methodologies are employed: MS profiling of biochemically segregated subcellular fractions [208], proximity-based protein labeling coupled with MS detection [209], and fluorescence microscopy-based visualization of protein distribution patterns [210]. These complementary techniques enable comprehensive mapping of the spatial proteome, elucidating the intricate relationships between protein distribution, organelle biology, and pathogenesis. Single-cell spatial technologies have emerged as powerful tools for unraveling the complexity of the tumor microenvironment (TME) by providing unprecedented insight into cellular heterogeneity, interactions, and spatial organization. Recent studies employing imaging mass cytometry (IMC) have shed light on the spatial landscapes of the TME in various cancer types, including lung adenocarcinoma [211, 212]. The use of these techniques to study the spatial distribution of exosomes and their protein cargo within the TME may provide unprecedented insights into the role of exosomes in lung cancer progression, metastasis, and immune modulation. Furthermore, tools such as SubCellBarCode, a robust and efficient mass spectrometry-based pipeline for proteome-wide mapping of protein subcellular localization [213], could be modified to map the subcellular origin and destination of exosomal proteins, thereby enhancing our understanding of the biogenesis and uptake of exosomes in lung cancer.

The integration of SCP with spatial proteomics offers a unique opportunity to understand cellular interactions and phenotypes at an unprecedented level of detail. Single-cell proteomics and spatial proteomics play complementary roles in the discovAIR project, contributing to the creation of a comprehensive Human Lung Cell Atlas [214]. Although significant progress has been made in SCP and spatial proteomics, the field of single-exosome proteomics is still in its infancy.

#### 6.2.4 Single-exosome proteomics

Current exosome proteomics studies have focused primarily on the population level rather than on individual exosome. However, single-exosome proteomics is the ultimate solution to alleviate the challenges posed by heterogeneity in exosome biomarker research. Single-exosome proteomics face unique challenges, such as extremely low protein content, and technical difficulties in isolating and enriching individual exosomes from different exosome subpopulations. While cell annotation in single-cell studies has matured, the annotation of various extracellular vesicles remains underdeveloped. Nevertheless, recent research progress has shown the potential feasibility of single-exosome proteomics. For instance, certain techniques used for single-cell isolation, such as microfluidics, can also be applied to single-exosome isolation. Droplet microarrays, a form of microfluidic technique, have emerged as one of the optimal platforms for pre-processing in single-cell proteomics [215]. This technology may also be applicable to single-exosome proteomics sample preparation. The encapsulated droplets provide compartments for efficient protein extraction and digestion, minimizing sample dilution and expediting the digestion process. In the near future, the continuous maturation of single-exosome isolation techniques coupled with cutting-edge Orbitrap Astral mass spectrometry may lead to significant breakthroughs in the field of single-exosome proteomics. Drawing insights from single-cell analysis techniques, methods can be adapted to study exosomes and other extracellular vesicles, elucidating the interplay between different exosome subpopulations and their synergistic or antagonistic effects on other extracellular vesicles. However, to our knowledge, such investigations remain unexplored.

In conclusion, emerging frontier proteomics technologies, including Orbitrap Astral mass spectrometry, multiplatform proteomic approaches, single-cell proteomics, and spatial proteomics, are paving the way for single-exosome proteomics in lung cancer research.

### 6.3 From single proteomics to multiomics

As omics research evolves toward multiomics, lung cancer exosome proteomics must also integrate with other omics methods to better understand the interplay and dynamic changes in various biomolecules carried by exosomes during cancer progression. However, this integration is challenging due to the heterogeneity of exosomes and the scale differences in proteogenomic measurements, thus, multiomics analysis does not always yield ideal results. Post-standardization, omics measurements may not align as expected due to discrepancies such as genetic mutations not leading to expression changes, RNA expression variations not resulting in actual protein changes, and post-translational modifications (PTMs) altering proteins without changing protein levels [216-219]. Such inconsistencies in omics measurements might be more pronounced at the exosomal level, necessitating a comprehensive assessment of all omics measurements. Recent reviews have summarized the current development of tools for implementing cross-variable approaches [220]. These integrative methods, by mapping changes directly onto transcriptomic, proteomic, or phosphoproteomic networks or by associating changes with pathways or response signatures, may enable cancer exosome proteogenomic research to uncover results greater than the sum of its parts, potentially further understanding the relationships between various biomolecules carried by exosomes and their roles in intercellular communication and cancer progression [221].

### 6.4 Translating exosome proteomics into clinical practice

#### 6.4.1 Dynamic profiling and longitudinal studies: capturing the exosome landscape

A significant research gap exists due to the lack of longitudinal and dynamic profiling studies, as most current data are gathered under static baseline conditions. Conducting longitudinal and dose-dependent studies to monitor exosome profile changes post-treatment or across disease stages will be essential for leveraging exosome proteomics in diagnosis, prognosis, and treatment [222]. Although logistically and analytically challenging, continuous improvements in the sensitivity and throughput of proteomic methods, such as microfluidics coupled with mass spectrometry, will enable high-resolution dynamic profiling, particularly in clinical settings with limited sample availability.

#### 6.4.2 The need for automated and integrated exosome isolation platforms

Developing automated, integrated, and cost-effective exosome analysis systems tailored for clinical use is essential for disseminating these techniques beyond experimental phases. Fully automated exosome isolation methods based on digital microfluidic platforms are garnering increased attention, as they minimize the impact of different isolation techniques and address the limitations of traditional methods, including lengthy procedures, high costs, and labor intensity. For example, Zhao *et al*. introduced an automated microfluidic system for efficient exosome isolation, demonstrating high diagnostic accuracy for lung cancer [223]. Similarly, Tong *et al*. showed a digital microfluidic technology for rapid EV pretreatment, enhancing liquid biopsies in lung cancer [224]. Other potential exosome isolation methods with clinical application value also warrant attention. For instance, surface plasmon resonance (SPR) platforms have been employed for the real-time, label-free profiling of clinically relevant exosomes (CREs), enabling the quantification of the proportion of CREs within the total exosome population [225]. Furthermore, the use of gold-loaded nanoporous ferric oxide nanozymes has facilitated the direct isolation and sensitive detection of exosomes, effectively eliminating the need for a pre-isolation step [226]. These advancements could bridge the gap between laboratory research and clinical application in lung cancer exosome proteomics. Studies have demonstrated the clinical application potential of digital microfluidic platforms for efficient exosome isolation and rapid EV pretreatment, enhancing lung cancer diagnosis through liquid biopsies.

#### 6.4.3 Simultaneous detection of multiple exosome-based biomarkers

Although individual biomarkers may provide valuable insights into specific aspects of lung cancer, their diagnostic and prognostic performance can be limited by the inherent variability in disease progression among patients. To overcome this limitation, the rational design of multiplexed assays that can simultaneously detect a panel of carefully selected exosome-based biomarkers is essential. However, the development of such assays requires a deep understanding of the molecular interplay between different biomarkers and their collective contribution to the disease phenotype. Moreover, the optimization of assay parameters, such as probe design, signal amplification, and data analysis, is crucial for ensuring the sensitivity, specificity, and reproducibility of the multiplexed detection system.

**Figure 3.**
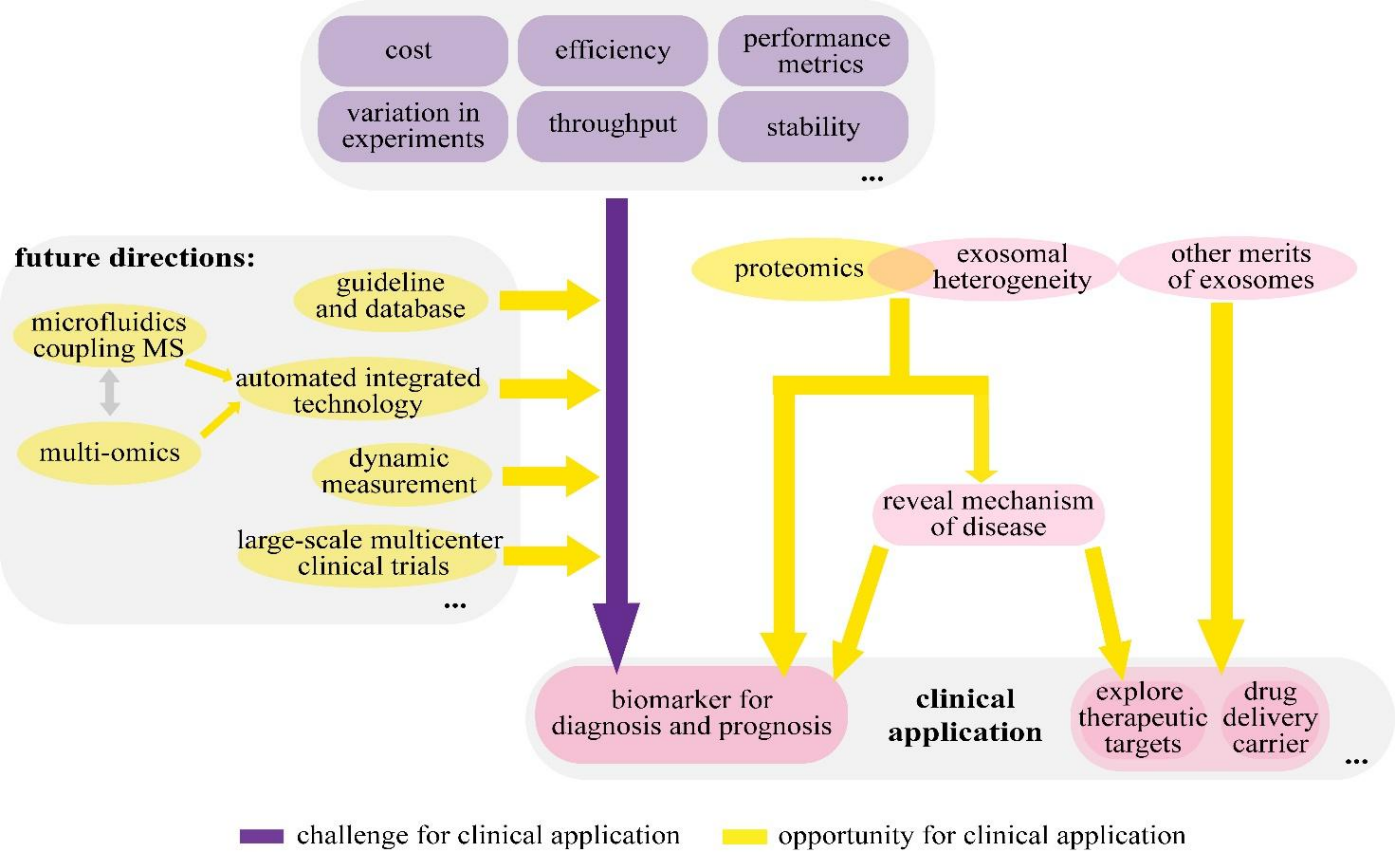
The opportunities and challenges in the clinical application of lung cancer exosome proteomics

#### 6.4.4 Validating biomarkers and facilitating clinical implementation

Numerous potential diagnostic, prognostic, and therapeutic biomarkers have been identified from exosome proteomics studies, but their clinical validity and utility remain largely unexplored. Large-scale, multicenter clinical trials are needed to rigorously validate the identified biomarkers across diverse patient populations and clinical settings. Recent reviews indicate ongoing progress in integrating exosome proteomics into clinical practice [227], with few exosome biomarkers currently approved for clinical use, such as the CLIA/FDA-approved ExoDx Prostate IntelliScore (EPI) test for prostate cancer [228]. More exosome biomarkers are expected to be validated and implemented in clinical practice.

In summary, the translation of exosome proteomics into clinical practice for lung cancer management holds great promise, but it also presents significant challenges. Dynamic profiling, automated isolation platforms, and multiplexed assays are crucial for capturing the exosome landscape and enhancing the diagnostic and prognostic performance of these tools. However, large-scale clinical trials are needed to rigorously validate the identified biomarkers. In summary, the opportunities and challenges in the clinical application of lung cancer exosome proteomics are briefly elucidated in Figure 3.

## Conclusion

In the rapidly evolving landscape of lung cancer research, exosomal proteomics is at the forefront, offering revolutionary insights into the molecular intricacies of cancer. Through the meticulous analysis of exosomes, researchers have identified a treasure trove of potential biomarkers and therapeutic targets that could fundamentally change our approach to the diagnosis, prognosis and treatment of lung cancer. The journey of exosomal proteomics from bench to bedside is laden with both promising and challenging, as evidenced by the remarkable advances in isolation techniques, multiomics integration, and the potential for clinical application highlighted in this review.

As we navigate the complexities of exosomal heterogeneity and strive to standardise research methodologies, it is clear that the path to clinical application is a collaborative endeavour, requiring the confluence of advanced technologies, rigorous clinical validation, and a nuanced understanding of the dynamic cancer exosome landscape. The promise of digital microfluidic platforms and single-exosome analysis in advancing our understanding of lung cancer at an unprecedented resolution underscores the innovative spirit driving this field forward. In conclusion, this review not only encapsulates the current state of knowledge but also charts a course for future research efforts. With each discovery, we move closer to unlocking the full potential of exosomal proteomics to transform lung cancer care, underscored by a commitment to overcome the challenges that lie ahead. The journey is complex, but the potential for impact on patient lives makes it a quest of unparalleled importance. The integration of exosomal proteomics into clinical practice will hopefully constitute a new era of precision medicine for lung cancer treatment.

## References

[1] SungH, FerlayJ, SiegelRL, LaversanneM, SoerjomataramI, JemalA, et al. (2021). Global Cancer Statistics 2020: GLOBOCAN Estimates of Incidence and Mortality Worldwide for 36 Cancers in 185 Countries. CA: a cancer journal for clinicians, 71:209-249.33538338 10.3322/caac.21660

[2] HowladerN, ForjazG, MooradianMJ, MezaR, KongCY, CroninKA, et al. (2020). The Effect of Advances in Lung-Cancer Treatment on Population Mortality. New England Journal of Medicine, 383:640-649.32786189 10.1056/NEJMoa1916623PMC8577315

[3] TaniguchiH, SenT, RudinCM (2020). Targeted Therapies and Biomarkers in Small Cell Lung Cancer. Frontiers in Oncology, 10:741.32509576 10.3389/fonc.2020.00741PMC7251180

[4] SchwendenweinA, MegyesfalviZ, BaranyN, ValkoZ, BugyikE, LangC, et al. (2021). Molecular profiles of small cell lung cancer subtypes: therapeutic implications. Molecular Therapy Oncolytics, 20:470-483.33718595 10.1016/j.omto.2021.02.004PMC7917449

[5] CaoW, ChenH-D, YuY-W, LiN, ChenW-Q (2021). Changing profiles of cancer burden worldwide and in China: a secondary analysis of the global cancer statistics 2020. Chinese Medical Journal, 134:783-791.33734139 10.1097/CM9.0000000000001474PMC8104205

[6] JacobsenMM, SilversteinSC, QuinnM, WaterstonLB, ThomasCA, BenneyanJC, et al. (2017). Timeliness of access to lung cancer diagnosis and treatment: A scoping literature review. Lung Cancer (Amsterdam, Netherlands), 112:156-164.29191588 10.1016/j.lungcan.2017.08.011

[7] SobueT, MoriyamaN, KanekoM, KusumotoM, KobayashiT, TsuchiyaR, et al. (2002). Screening for Lung Cancer With Low-Dose Helical Computed Tomography: Anti-Lung Cancer Association Project. Journal of Clinical Oncology, 20:911-920.11844811 10.1200/JCO.2002.20.4.911

[8] ToyodaY, NakayamaT, KusunokiY, IsoH, SuzukiT (2008). Sensitivity and specificity of lung cancer screening using chest low-dose computed tomography. British Journal of Cancer, 98:1602-1607.18475292 10.1038/sj.bjc.6604351PMC2391122

[9] LiZ-B, ChenD-D, HeQ-J, LiL, ZhouG, FuY-M, et al. (2021). The LAC Score Indicates Significant Fibrosis in Patients With Chronic Drug-Induced Liver Injury: A Large Biopsy-Based Study. Frontiers in Pharmacology, 12:734090.34483945 10.3389/fphar.2021.734090PMC8416439

[10] BeckerA, ThakurBK, WeissJM, KimHS, PeinadoH, LydenD (2016). Extracellular Vesicles in Cancer: Cell-to-Cell Mediators of Metastasis. Cancer Cell, 30:836-848.27960084 10.1016/j.ccell.2016.10.009PMC5157696

[11] ZhangY-C, ZhouQ, WuY-L (2017). The emerging roles of NGS-based liquid biopsy in non-small cell lung cancer. Journal of Hematology & Oncology, 10:167.29061113 10.1186/s13045-017-0536-6PMC5654124

[12] LinS, YuZ, ChenD, WangZ, MiaoJ, LiQ, et al. (2020). Progress in Microfluidics-Based Exosome Separation and Detection Technologies for Diagnostic Applications. Small, 16:e1903916.31663295 10.1002/smll.201903916

[13] KalluriR, LeBleuVS (2020). The biology, function, and biomedical applications of exosomes. Science (New York, N.Y.), 367:eaau6977.32029601 10.1126/science.aau6977PMC7717626

[14] DingZ, WangN, JiN, ChenZ-S (2022). Proteomics technologies for cancer liquid biopsies. Molecular Cancer, 21:53.35168611 10.1186/s12943-022-01526-8PMC8845389

[15] LaiJJ, ChauZL, ChenS-Y, HillJJ, KorpanyKV, LiangN-W, et al. (2022). Exosome Processing and Characterization Approaches for Research and Technology Development. Advanced Science (Weinheim, Baden-Wurttemberg, Germany), 9:e2103222.35332686 10.1002/advs.202103222PMC9130923

[16] BebelmanMP, SmitMJ, PegtelDM, BaglioSR (2018). Biogenesis and function of extracellular vesicles in cancer. Pharmacology & Therapeutics, 188:1-11.29476772 10.1016/j.pharmthera.2018.02.013

[17] WollertT, HurleyJH (2010). Molecular mechanism of multivesicular body biogenesis by ESCRT complexes. Nature, 464:864-869.20305637 10.1038/nature08849PMC2851844

[18] C T, M O, E S (2009). Membrane vesicles as conveyors of immune responses. Nature reviews. Immunology, 9. 10.1038/nri2567 19498381

[19] DoyleLM, WangMZ (2019). Overview of Extracellular Vesicles, Their Origin, Composition, Purpose, and Methods for Exosome Isolation and Analysis. Cells, 8:727.31311206 10.3390/cells8070727PMC6678302

[20] Yáñez-MóM, SiljanderPR-M, AndreuZ, ZavecAB, BorràsFE, BuzasEI, et al. (2015). Biological properties of extracellular vesicles and their physiological functions. Journal of Extracellular Vesicles, 4:27066.25979354 10.3402/jev.v4.27066PMC4433489

[21] LiP, KaslanM, LeeSH, YaoJ, GaoZ (2017). Progress in Exosome Isolation Techniques. Theranostics, 7:789-804.28255367 10.7150/thno.18133PMC5327650

[22] AfridiWA, StrachanS, KasetsirikulS, PannuAS, SodaN, GoughD, et al. (2023). Potential Avenues for Exosomal Isolation and Detection Methods to Enhance Small-Cell Lung Cancer Analysis. ACS measurement science au, 3:143-161.37360040 10.1021/acsmeasuresciau.2c00068PMC10288614

[23] Boriachek K, Islam MN, Möller A, Salomon C, Nguyen N-T, Hossain MSA, et al. (2018). Biological Functions and Current Advances in Isolation and Detection Strategies for Exosome Nanovesicles. Small (Weinheim an Der Bergstrasse, Germany), 14. 10.1002/smll.201702153 29282861

[24] MullerL, HongC-S, StolzDB, WatkinsSC, WhitesideTL (2014). Isolation of biologically-active exosomes from human plasma. Journal of Immunological Methods, 411:55-65.24952243 10.1016/j.jim.2014.06.007PMC4260336

[25] KingSL, JoshiHJ, SchjoldagerKT, HalimA, MadsenTD, DziegielMH, et al. (2017). Characterizing the O-glycosylation landscape of human plasma, platelets, and endothelial cells. Blood advances, 1:429-442.29296958 10.1182/bloodadvances.2016002121PMC5738978

[26] DoyleLM, WangMZ (2019). Overview of Extracellular Vesicles, Their Origin, Composition, Purpose, and Methods for Exosome Isolation and Analysis. Cells, 8.31311206 10.3390/cells8070727PMC6678302

[27] VogelR, CoumansFAW, MaltesenRG, BöingAN, BonningtonKE, BroekmanML, et al. (2016). A standardized method to determine the concentration of extracellular vesicles using tunable resistive pulse sensing. Journal of Extracellular Vesicles, 5:31242.27680301 10.3402/jev.v5.31242PMC5040823

[28] MullerL, HongCS, StolzDB, WatkinsSC, WhitesideTL (2014). Isolation of biologically-active exosomes from human plasma. J Immunol Methods, 411:55-65.24952243 10.1016/j.jim.2014.06.007PMC4260336

[29] JiaY, YuL, MaT, XuW, QianH, SunY, et al. (2022). Small extracellular vesicles isolation and separation: Current techniques, pending questions and clinical applications. Theranostics, 12:6548-6575.36185597 10.7150/thno.74305PMC9516236

[30] LiY, WangJ, ChenW, LuH, ZhangY (2023). Comprehensive review of MS-based studies on N-glycoproteome and N-glycome of extracellular vesicles. Proteomics:e2300065.37474487 10.1002/pmic.202300065

[31] BoriachekK, IslamMN, GopalanV, LamAK, NguyenN-T, ShiddikyMJA (2017). Quantum dot-based sensitive detection of disease specific exosome in serum. The Analyst, 142:2211-2219.28534915 10.1039/c7an00672a

[32] ShirejiniSZ, InciF (2022). The Yin and Yang of exosome isolation methods: conventional practice, microfluidics, and commercial kits. Biotechnol Adv, 54:107814.34389465 10.1016/j.biotechadv.2021.107814

[33] YangC, KongGL, WangLW, ZhangLX, ZhaoMH (2019). Big data in nephrology: Are we ready for the change? Nephrology, 24:1097-1102.31314170 10.1111/nep.13636

[34] SooCY, SongY, ZhengY, CampbellEC, RichesAC, Gunn-MooreF, et al. (2012). Nanoparticle tracking analysis monitors microvesicle and exosome secretion from immune cells. Immunology, 136:192-197.22348503 10.1111/j.1365-2567.2012.03569.xPMC3403268

[35] SitarS, KejžarA, PahovnikD, KogejK, Tušek-ŽnidaričM, LenassiM, et al. (2015). Size characterization and quantification of exosomes by asymmetrical-flow field-flow fractionation. Anal Chem, 87:9225-9233.26291637 10.1021/acs.analchem.5b01636

[36] ShuklaS, CurrimF, SinghR (2023). Do different exosome biogenesis pathways and selective cargo enrichment contribute to exosomal heterogeneity? Biol Cell, 115:e2200116.37179461 10.1111/boc.202200116

[37] PhillipsW, WillmsE, HillAF (2021). Understanding extracellular vesicle and nanoparticle heterogeneity: Novel methods and considerations. Proteomics, 21:e2000118.33857352 10.1002/pmic.202000118PMC8365743

[38] BarrancoI, PadillaL, ParrillaI, Álvarez-BarrientosA, Pérez-PatiñoC, PeñaFJ, et al. (2019). Extracellular vesicles isolated from porcine seminal plasma exhibit different tetraspanin expression profiles. Sci Rep, 9:11584.31399634 10.1038/s41598-019-48095-3PMC6689046

[39] LyuTS, AhnY, ImYJ, KimSS, LeeKH, KimJ, et al. (2021). The characterization of exosomes from fibrosarcoma cell and the useful usage of Dynamic Light Scattering (DLS) for their evaluation. PLoS One, 16:e0231994.33497388 10.1371/journal.pone.0231994PMC7837462

[40] MatsudaA, KunoA, YoshidaM, WagatsumaT, SatoT, MiyagishiM, et al. (2020). Comparative Glycomic Analysis of Exosome Subpopulations Derived from Pancreatic Cancer Cell Lines. J Proteome Res, 19:2516-2524.32338917 10.1021/acs.jproteome.0c00200

[41] Dimitrakopoulos FI, Kottorou AE, Rodgers K, Sherwood JT, Koliou GA, Lee B, et al. (2021). Clinical Significance of Plasma CD9-Positive Exosomes in HIV Seronegative and Seropositive Lung Cancer Patients. Cancers(Basel), 13. 10.3390/cancers13205193 PMC8533968 34680341

[42] HuangHM, LiHX (2021). Tumor heterogeneity and the potential role of liquid biopsy in bladder cancer. Cancer Commun (Lond), 41:91-108.33377623 10.1002/cac2.12129PMC7896752

[43] ValenciaK, MontuengaLM (2021). Exosomes in Liquid Biopsy: The Nanometric World in the Pursuit of Precision Oncology. Cancers (Basel), 13.33946893 10.3390/cancers13092147PMC8124368

[44] AnJ, YangH, YangE, ChungS, KimDY, JouI, et al. (2021). Dying neurons conduct repair processes in the injured brain through osteopontin expression in cooperation with infiltrated blood monocytes. Glia, 69:1037-1052.33300228 10.1002/glia.23947

[45] YunusovaNV, ZambalovaEA, PatyshevaMR, KolegovaES, Afanas'evSG, CheremisinaOV, et al. (2021). Exosomal Protease Cargo as Prognostic Biomarker in Colorectal Cancer. Asian Pac J Cancer Prev, 22:861-869.33773551 10.31557/APJCP.2021.22.3.861PMC8286660

[46] Chao CT, Chiang CK, Hung KY (2023). Extracellular MicroRNAs as Potential Biomarkers for Frail Kidney Phenotype: Progresses and Precautions. Aging Dis. 10.14336/AD.2023.0818 PMC11272190 37611904

[47] RenH, GuoZ, LiuY, SongC (2022). Stem Cell-derived Exosomal MicroRNA as Therapy for Vascular Age-related Diseases. Aging Dis, 13:852-867.35656114 10.14336/AD.2021.1110PMC9116915

[48] WuSC, KuoPJ, RauCS, WuYC, WuCJ, LuTH, et al. (2021). Subpopulations of exosomes purified via different exosomal markers carry different microRNA contents. Int J Med Sci, 18:1058-1066.33456364 10.7150/ijms.52768PMC7807189

[49] KarimiN, DalirfardoueiR, DiasT, LötvallJ, LässerC (2022). Tetraspanins distinguish separate extracellular vesicle subpopulations in human serum and plasma - Contributions of platelet extracellular vesicles in plasma samples. J Extracell Vesicles, 11:e12213.35524458 10.1002/jev2.12213PMC9077141

[50] ChiangCY, ChenC (2019). Toward characterizing extracellular vesicles at a single-particle level. J Biomed Sci, 26:9.30646920 10.1186/s12929-019-0502-4PMC6332877

[51] SinghK, NalabotalaR, KooKM, BoseS, NayakR, ShiddikyMJA (2021). Separation of distinct exosome subpopulations: isolation and characterization approaches and their associated challenges. The Analyst, 146:3731-3749.33988193 10.1039/d1an00024a

[52] PhanTH, DivakarlaSK, YeoJH, LeiQ, TharkarP, PansaniTN, et al. (2021). New Multiscale Characterization Methodology for Effective Determination of Isolation-Structure-Function Relationship of Extracellular Vesicles. Front Bioeng Biotechnol, 9:669537.34164385 10.3389/fbioe.2021.669537PMC8215393

[53] MaX, ChenZ, ChenW, ChenZ, MengX (2024). Exosome subpopulations: The isolation and the functions in diseases. Gene, 893:147905.37844851 10.1016/j.gene.2023.147905

[54] Gu Y, Chen C, Mao Z, Bachman H, Becker R, Rufo J, et al. (2021). Acoustofluidic centrifuge for nanoparticle enrichment and separation. Sci Adv, 7. 10.1126/sciadv.abc0467 PMC7775782 33523836

[55] ZhangH, FreitasD, KimHS, FabijanicK, LiZ, ChenH, et al. (2018). Identification of distinct nanoparticles and subsets of extracellular vesicles by asymmetric flow field-flow fractionation. Nat Cell Biol, 20:332-343.29459780 10.1038/s41556-018-0040-4PMC5931706

[56] KowalJ, ArrasG, ColomboM, JouveM, MorathJP, Primdal-BengtsonB, et al. (2016). Proteomic comparison defines novel markers to characterize heterogeneous populations of extracellular vesicle subtypes. Proc Natl Acad Sci U S A, 113:E968-977.26858453 10.1073/pnas.1521230113PMC4776515

[57] TamkovichSN, YunusovaNV, TugutovaE, SomovAK, ProskuraKV, KolomietsLA, et al. (2019). Protease Cargo in Circulating Exosomes of Breast Cancer and Ovarian Cancer Patients. Asian Pac J Cancer Prev, 20:255-262.30678441 10.31557/APJCP.2019.20.1.255PMC6485591

[58] ZhuJ, ZhangJ, JiX, TanZ, LubmanDM (2021). Column-based Technology for CD9-HPLC Immunoaffinity Isolation of Serum Extracellular Vesicles. J Proteome Res, 20:4901-4911.34473505 10.1021/acs.jproteome.1c00549PMC8496948

[59] WangY, WangS, ChenA, WangR, LiL, FangX (2022). Efficient exosome subpopulation isolation and proteomic profiling using a Sub-ExoProfile chip towards cancer diagnosis and treatment. Analyst, 147:4237-4248.36062905 10.1039/d2an01268e

[60] ShenX, YangY, ChenY, ZhouC, ZhaoX, LiN, et al. (2022). Evaluation of EpCAM-specific exosomal lncRNAs as potential diagnostic biomarkers for lung cancer using droplet digital PCR. J Mol Med (Berl), 100:87-100.34651202 10.1007/s00109-021-02145-4

[61] HatanoA, ChibaH, MoesaHA, TaniguchiT, NagaieS, YamanegiK, et al. (2011). CELLPEDIA: a repository for human cell information for cell studies and differentiation analyses. Database (Oxford), 2011:bar046.22039163 10.1093/database/bar046PMC3204613

[62] Regev A, Teichmann SA, Lander ES, Amit I, Benoist C, Birney E, et al. (2017). The Human Cell Atlas. Elife, 6. 10.7554/eLife.27041 PMC5762154 29206104

[63] TkachM, KowalJ, ZucchettiAE, EnserinkL, JouveM, LankarD, et al. (2017). Qualitative differences in T-cell activation by dendritic cell-derived extracellular vesicle subtypes. Embo j, 36:3012-3028.28923825 10.15252/embj.201696003PMC5641679

[64] Del CondeI, ShrimptonCN, ThiagarajanP, LópezJA (2005). Tissue-factor-bearing microvesicles arise from lipid rafts and fuse with activated platelets to initiate coagulation. Blood, 106:1604-1611.15741221 10.1182/blood-2004-03-1095

[65] AntonucciF, TurolaE, RigantiL, CaleoM, GabrielliM, PerrottaC, et al. (2012). Microvesicles released from microglia stimulate synaptic activity via enhanced sphingolipid metabolism. Embo j, 31:1231-1240.22246184 10.1038/emboj.2011.489PMC3297996

[66] ChanteloupG, CordonnierM, IsambertN, BertautA, HervieuA, HennequinA, et al. (2020). Monitoring HSP70 exosomes in cancer patients' follow up: a clinical prospective pilot study. J Extracell Vesicles, 9:1766192.32595915 10.1080/20013078.2020.1766192PMC7301715

[67] HongW, XueM, JiangJ, ZhangY, GaoX (2020). Circular RNA circ-CPA4/ let-7 miRNA/PD-L1 axis regulates cell growth, stemness, drug resistance and immune evasion in non-small cell lung cancer (NSCLC). J Exp Clin Cancer Res, 39:149.32746878 10.1186/s13046-020-01648-1PMC7397626

[68] JinX, ChenY, ChenH, FeiS, ChenD, CaiX, et al. (2017). Evaluation of Tumor-Derived Exosomal miRNA as Potential Diagnostic Biomarkers for Early-Stage Non-Small Cell Lung Cancer Using Next-Generation Sequencing. Clin Cancer Res, 23:5311-5319.28606918 10.1158/1078-0432.CCR-17-0577

[69] Diaz-HidalgoL, AltuntasS, RossinF, D'ElettoM, MarsellaC, FarraceMG, et al. (2016). Transglutaminase type 2-dependent selective recruitment of proteins into exosomes under stressful cellular conditions. Biochim Biophys Acta, 1863:2084-2092.27169926 10.1016/j.bbamcr.2016.05.005

[70] JiaoY, LuW, XuP, ShiH, ChenD, ChenY, et al. (2021). Hepatocyte-derived exosome may be as a biomarker of liver regeneration and prognostic valuation in patients with acute-on-chronic liver failure. Hepatol Int, 15:957-969.34232468 10.1007/s12072-021-10217-3

[71] LuH, ZhangY, XiongS, ZhouY, XiaoL, MaY, et al. (2021). Modulatory Role of Silver Nanoparticles and Mesenchymal Stem Cell-Derived Exosome-Modified Barrier Membrane on Macrophages and Osteogenesis. Front Chem, 9:699802.34409016 10.3389/fchem.2021.699802PMC8365089

[72] HsiaoYP, ChenC, LeeCM, ChenPY, ChungWH, WangYP, et al. (2021). Differences in the Quantity and Composition of Extracellular Vesicles in the Aqueous Humor of Patients with Retinal Neovascular Diseases. Diagnostics (Basel), 11.34359359 10.3390/diagnostics11071276PMC8306174

[73] NikamRV, GowthamM, MorePS, ShindeAS (2023). Current and emerging prospects in the psoriatic treatment. Int Immunopharmacol, 120:110331.37210912 10.1016/j.intimp.2023.110331

[74] Jia H, Liu T, Yang Q, Zheng H, Fu S, Hong J, et al. (2023). Tumor-Derived PD-L1(+) Exosomes with Natural Inflammation Tropism for Psoriasis-Targeted Treatment. Bioconjug Chem. 10.1021/acs.bioconjchem.3c00129 37036892

[75] KimH, ZhaoQ, BarredaH, KaurG, HaiB, ChoiJM, et al. (2021). Identification of Molecules Responsible for Therapeutic Effects of Extracellular Vesicles Produced from iPSC-Derived MSCs on Sjögren's Syndrome. Aging Dis, 12:1409-1422.34527418 10.14336/AD.2021.0621PMC8407887

[76] XueC, LiX, BaL, ZhangM, YangY, GaoY, et al. (2021). MSC-Derived Exosomes can Enhance the Angiogenesis of Human Brain MECs and Show Therapeutic Potential in a Mouse Model of Parkinson's Disease. Aging Dis, 12:1211-1222.34341703 10.14336/AD.2020.1221PMC8279521

[77] ConradDH, GoyetteJ, ThomasPS (2008). Proteomics as a Method for Early Detection of Cancer: A Review of Proteomics, Exhaled Breath Condensate, and Lung Cancer Screening. Journal of General Internal Medicine, 23:78-84.18095050 10.1007/s11606-007-0411-1PMC2150625

[78] YangW, ShahP, HuY, Toghi EshghiS, SunS, LiuY, et al. (2017). Comparison of Enrichment Methods for Intact N- and O-Linked Glycopeptides Using Strong Anion Exchange and Hydrophilic Interaction Liquid Chromatography. Analytical chemistry, 89:11193-11197.29016103 10.1021/acs.analchem.7b03641PMC5850954

[79] RossPL, HuangYN, MarcheseJN, WilliamsonB, ParkerK, HattanS, et al. (2004). Multiplexed Protein Quantitation in Saccharomyces cerevisiae Using Amine-reactive Isobaric Tagging Reagents. Molecular & Cellular Proteomics, 3:1154-1169.15385600 10.1074/mcp.M400129-MCP200

[80] CheungCHY, JuanH-F (2017). Quantitative proteomics in lung cancer. Journal of Biomedical Science, 24:37.28615068 10.1186/s12929-017-0343-yPMC5470322

[81] KarUK, SimonianM, WhiteleggeJP (2017). Integral membrane proteins: bottom-up, top-down and structural proteomics. Expert Rev Proteomics, 14:715-723.28737967 10.1080/14789450.2017.1359545PMC6310004

[82] ChenX, WeiS, JiY, GuoX, YangF (2015). Quantitative proteomics using SILAC: Principles, applications, and developments. Proteomics, 15:3175-3192.26097186 10.1002/pmic.201500108

[83] WieseS, ReidegeldKA, MeyerHE, WarscheidB (2007). Protein labeling by iTRAQ: A new tool for quantitative mass spectrometry in proteome research. PROTEOMICS, 7:340-350.17177251 10.1002/pmic.200600422

[84] MoulderR, BhosaleSD, GoodlettDR, LahesmaaR (2018). Analysis of the plasma proteome using iTRAQ and TMT-based Isobaric labeling. Mass Spectrom Rev, 37:583-606.29120501 10.1002/mas.21550

[85] Gasparri R, Sedda G, Noberini R, Bonaldi T, Spaggiari L (2020). Clinical Application of Mass Spectrometry-Based Proteomics in Lung Cancer Early Diagnosis. PROTEOMICS - Clinical Applications, 14:1900138. 10.1002/prca.201900138 32418314

[86] Fernández-CostaC, Martínez-BartoloméS, McClatchyDB, SaviolaAJ, YuN-K, YatesJR (2020). Impact of the Identification Strategy on the Reproducibility of the DDA and DIA Results. Journal of Proteome Research, 19:3153-3161.32510229 10.1021/acs.jproteome.0c00153PMC7898222

[87] KrasnyL, HuangPH (2021). Data-independent acquisition mass spectrometry (DIA-MS) for proteomic applications in oncology. Molecular Omics, 17:29-42.33034323 10.1039/d0mo00072h

[88] ChenC, HouJ, TannerJJ, ChengJ (2020). Bioinformatics Methods for Mass Spectrometry-Based Proteomics Data Analysis. Int J Mol Sci, 21.32326049 10.3390/ijms21082873PMC7216093

[89] EngJK, McCormackAL, YatesJR (1994). An approach to correlate tandem mass spectral data of peptides with amino acid sequences in a protein database. J Am Soc Mass Spectrom, 5:976-989.24226387 10.1016/1044-0305(94)80016-2

[90] DancíkV, AddonaTA, ClauserKR, VathJE, PevznerPA (1999). De novo peptide sequencing via tandem mass spectrometry. J Comput Biol, 6:327-342.10582570 10.1089/106652799318300

[91] EngJK, FischerB, GrossmannJ, MaccossMJ (2008). A fast SEQUEST cross correlation algorithm. J Proteome Res, 7:4598-4602.18774840 10.1021/pr800420s

[92] PerkinsDN, PappinDJ, CreasyDM, CottrellJS (1999). Probability-based protein identification by searching sequence databases using mass spectrometry data. Electrophoresis, 20:3551-3567.10612281 10.1002/(SICI)1522-2683(19991201)20:18<3551::AID-ELPS3551>3.0.CO;2-2

[93] SavitskiMM, NielsenML, ZubarevRA (2006). ModifiComb, a new proteomic tool for mapping substoichiometric post-translational modifications, finding novel types of modifications, and fingerprinting complex protein mixtures. Mol Cell Proteomics, 5:935-948.16439352 10.1074/mcp.T500034-MCP200

[94] PercheyRT, ToniniL, TosoliniM, FourniéJJ, LopezF, BessonA, et al. (2019). PTMselect: optimization of protein modifications discovery by mass spectrometry. Sci Rep, 9:4181.30862887 10.1038/s41598-019-40873-3PMC6414543

[95] RudolphJD, CoxJ (2019). A Network Module for the Perseus Software for Computational Proteomics Facilitates Proteome Interaction Graph Analysis. J Proteome Res, 18:2052-2064.30931570 10.1021/acs.jproteome.8b00927PMC6578358

[96] WiśniewskiJR, MannM (2016). A Proteomics Approach to the Protein Normalization Problem: Selection of Unvarying Proteins for MS-Based Proteomics and Western Blotting. J Proteome Res, 15:2321-2326.27297043 10.1021/acs.jproteome.6b00403

[97] McHughML (2011). Multiple comparison analysis testing in ANOVA. Biochem Med (Zagreb), 21:203-209.22420233 10.11613/bm.2011.029

[98] KammersK, ColeRN, TiengweC, RuczinskiI (2015). Detecting Significant Changes in Protein Abundance. EuPA Open Proteom, 7:11-19.25821719 10.1016/j.euprot.2015.02.002PMC4373093

[99] ReelPS, ReelS, PearsonE, TruccoE, JeffersonE (2021). Using machine learning approaches for multi-omics data analysis: A review. Biotechnol Adv, 49:107739.33794304 10.1016/j.biotechadv.2021.107739

[100] Hindson J (2022). Proteomics and machine-learning models for alcohol-related liver disease biomarkers. Nat Rev Gastroenterol Hepatol, 19:488. 10.1038/s41575-022-00655-1 35773390

[101] TsukitaK, Sakamaki-TsukitaH, KaiserS, ZhangL, MessaM, Serrano-FernandezP, et al. (2023). High-Throughput CSF Proteomics and Machine Learning to Identify Proteomic Signatures for Parkinson Disease Development and Progression. Neurology, 101:e1434-e1447.37586882 10.1212/WNL.0000000000207725PMC10573147

[102] DennisGJr., ShermanBT, HosackDA, YangJ, GaoW, LaneHC, et al. (2003). DAVID: Database for Annotation, Visualization, and Integrated Discovery. Genome Biol, 4:P3.12734009

[103] SzklarczykD, KirschR, KoutrouliM, NastouK, MehryaryF, HachilifR, et al. (2023). The STRING database in 2023: protein-protein association networks and functional enrichment analyses for any sequenced genome of interest. Nucleic Acids Res, 51:D638-d646.36370105 10.1093/nar/gkac1000PMC9825434

[104] BauerA, KusterB (2003). Affinity purification-mass spectrometry. Powerful tools for the characterization of protein complexes. Eur J Biochem, 270:570-578.12581197 10.1046/j.1432-1033.2003.03428.x

[105] BenabdelkamelH, MasoodA, OklaM, Al-NaamiMY, AlfaddaAA (2019). A Proteomics-Based Approach Reveals Differential Regulation of Urine Proteins between Metabolically Healthy and Unhealthy Obese Patients. Int J Mol Sci, 20.31623319 10.3390/ijms20194905PMC6801506

[106] PieroniL, IavaroneF, OlianasA, GrecoV, DesiderioC, MartelliC, et al. (2020). Enrichments of post-translational modifications in proteomic studies. J Sep Sci, 43:313-336.31631532 10.1002/jssc.201900804

[107] MischnikM, SaccoF, CoxJ, SchneiderHC, SchäferM, HendlichM, et al. (2016). IKAP: A heuristic framework for inference of kinase activities from Phosphoproteomics data. Bioinformatics, 32:424-431.26628587 10.1093/bioinformatics/btv699

[108] WiredjaDD, KoyutürkM, ChanceMR (2017). The KSEA App: a web-based tool for kinase activity inference from quantitative phosphoproteomics. Bioinformatics, 33:3489-3491.28655153 10.1093/bioinformatics/btx415PMC5860163

[109] WirbelJ, CutillasP, Saez-RodriguezJ (2018). Phosphoproteomics-Based Profiling of Kinase Activities in Cancer Cells. Methods Mol Biol, 1711:103-132.29344887 10.1007/978-1-4939-7493-1_6PMC6126574

[110] KalluriR, LeBleuVS (2020). The biology, function, and biomedical applications of exosomes. Science, 367.32029601 10.1126/science.aau6977PMC7717626

[111] ZhangL, YuD (2019). Exosomes in cancer development, metastasis, and immunity. Biochim Biophys Acta Rev Cancer, 1871:455-468.31047959 10.1016/j.bbcan.2019.04.004PMC6542596

[112] LouP, LiuS, WangY, LvK, ZhouX, LiL, et al. (2023). Neonatal-Tissue-Derived Extracellular Vesicle Therapy (NEXT): A Potent Strategy for Precision Regenerative Medicine. Adv Mater, 35:e2300602.37148469 10.1002/adma.202300602

[113] CastilloJ, BernardV, San LucasFA, AllensonK, CapelloM, KimDU, et al. (2018). Surfaceome profiling enables isolation of cancer-specific exosomal cargo in liquid biopsies from pancreatic cancer patients. Annals of Oncology: Official Journal of the European Society for Medical Oncology, 29:223-229.29045505 10.1093/annonc/mdx542PMC6248757

[114] CvjetkovicA, JangSC, KonečnáB, HöögJL, SihlbomC, LässerC, et al. (2016). Detailed Analysis of Protein Topology of Extracellular Vesicles-Evidence of Unconventional Membrane Protein Orientation. Scientific Reports, 6:36338.27821849 10.1038/srep36338PMC5099568

[115] HoshinoA, Costa-SilvaB, ShenT-L, RodriguesG, HashimotoA, Tesic MarkM, et al. (2015). Tumour exosome integrins determine organotropic metastasis. Nature, 527:329-335.26524530 10.1038/nature15756PMC4788391

[116] BlancL, VidalM (2018). New insights into the function of Rab GTPases in the context of exosomal secretion. Small GTPases, 9:95-106.28135905 10.1080/21541248.2016.1264352PMC5902209

[117] PhuyalS, HessvikNP, SkotlandT, SandvigK, LlorenteA (2014). Regulation of exosome release by glycosphingolipids and flotillins. The FEBS journal, 281:2214-2227.24605801 10.1111/febs.12775

[118] MazurovD, BarbashovaL, FilatovA (2013). Tetraspanin protein CD9 interacts with metalloprotease CD10 and enhances its release via exosomes. The FEBS journal, 280:1200-1213.23289620 10.1111/febs.12110

[119] LynchS, SantosSG, CampbellEC, NimmoAMS, BottingC, PrescottA, et al. (2009). Novel MHC class I structures on exosomes. Journal of Immunology (Baltimore, Md.: 1950), 183:1884-1891.19596992 10.4049/jimmunol.0900798

[120] J C, V B, Fa SL, K A, M C, Du K, et al. (2018). Surfaceome profiling enables isolation of cancer-specific exosomal cargo in liquid biopsies from pancreatic cancer patients. Annals of oncology : official journal of the European Society for Medical Oncology, 29. 10.1093/annonc/mdx542 PMC6248757 29045505

[121] van NielG, D'AngeloG, RaposoG (2018). Shedding light on the cell biology of extracellular vesicles. Nature Reviews. Molecular Cell Biology, 19:213-228.29339798 10.1038/nrm.2017.125

[122] Colombo M, Raposo G, Théry C (2014). Biogenesis, secretion, and intercellular interactions of exosomes and other extracellular vesicles. Annual Review of Cell and Developmental Biology, 30:255-289. 10.1146/annurev-cellbio-101512-122326 25288114

[123] ZhengX, ChenF, ZhangQ, LiuY, YouP, SunS, et al. (2017). Salivary exosomal PSMA7: a promising biomarker of inflammatory bowel disease. Protein & Cell, 8:686-695.28523434 10.1007/s13238-017-0413-7PMC5563283

[124] QiB, KongL, LaiX, WangL, LiuF, JiW, et al. (2023). Plasma exosome proteomics reveals the pathogenesis mechanism of post-stroke cognitive impairment. Aging, 15:4334-4362.37211381 10.18632/aging.204738PMC10258006

[125] BruschiM, GranataS, SantucciL, CandianoG, FabrisA, AntonucciN, et al. (2019). Proteomic Analysis of Urinary Microvesicles and Exosomes in Medullary Sponge Kidney Disease and Autosomal Dominant Polycystic Kidney Disease. Clinical journal of the American Society of Nephrology: CJASN, 14:834-843.31018934 10.2215/CJN.12191018PMC6556712

[126] LiuT, LiuN, WangY, LiT, ZhangM (2023). Differential expression of coagulation pathway-related proteins in diabetic urine exosomes. Cardiovascular Diabetology, 22:145.37349729 10.1186/s12933-023-01887-4PMC10288686

[127] LiL, SongX, ChenG, ZhangZ, ZhengB, ZhangQ, et al. (2023). Plasma exosomal protein PLG and SERPINA1 in colorectal cancer diagnosis and coagulation abnormalities. Journal of Cancer Research and Clinical Oncology, 149:8507-8519.37093347 10.1007/s00432-023-04776-1PMC11796920

[128] DingX-Q, WangZ-Y, XiaD, WangR-X, PanX-R, TongJ-H (2020). Proteomic Profiling of Serum Exosomes From Patients With Metastatic Gastric Cancer. Frontiers in Oncology, 10:1113.32754443 10.3389/fonc.2020.01113PMC7367030

[129] FengX, JiaS, AliMM, ZhangG, LiD, TaoWA, et al. (2023). Proteomic Discovery and Array-Based Validation of Biomarkers from Urinary Exosome by Supramolecular Probe. Journal of Proteome Research, 22:2516-2524.37126797 10.1021/acs.jproteome.3c00063

[130] TomiyamaE, MatsuzakiK, FujitaK, ShiromizuT, NarumiR, JingushiK, et al. (2021). Proteomic analysis of urinary and tissue-exudative extracellular vesicles to discover novel bladder cancer biomarkers. Cancer Science, 112:2033-2045.33721374 10.1111/cas.14881PMC8088963

[131] IliukA, WuX, LiL, SunJ, HadisuryaM, BorisRS, et al. (2020). Plasma-Derived Extracellular Vesicle Phosphoproteomics through Chemical Affinity Purification. Journal of Proteome Research, 19:2563-2574.32396726 10.1021/acs.jproteome.0c00151PMC7479851

[132] SongY, WangM, TongH, TanY, HuX, WangK, et al. (2021). Plasma exosomes from endometrial cancer patients contain LGALS3BP to promote endometrial cancer progression. Oncogene, 40:633-646.33208911 10.1038/s41388-020-01555-x

[133] ZhangW, OuX, WuX (2019). Proteomics profiling of plasma exosomes in epithelial ovarian cancer: A potential role in the coagulation cascade, diagnosis and prognosis. International Journal of Oncology, 54:1719-1733.30864689 10.3892/ijo.2019.4742PMC6438431

[134] WangT, ZhengSL, LiuL, VoglmeirJ (2019). Development of a colorimetric PNGase activity assay. Carbohydr Res, 472:58-64.30476755 10.1016/j.carres.2018.11.007

[135] WuC-H, SilversCR, MessingEM, LeeY-F (2019). Bladder cancer extracellular vesicles drive tumorigenesis by inducing the unfolded protein response in endoplasmic reticulum of nonmalignant cells. The Journal of Biological Chemistry, 294:3207-3218.30593508 10.1074/jbc.RA118.006682PMC6398136

[136] RodriguesG, HoshinoA, KenificCM, MateiIR, SteinerL, FreitasD, et al. (2019). Tumour exosomal CEMIP protein promotes cancer cell colonization in brain metastasis. Nature Cell Biology, 21:1403-1412.31685984 10.1038/s41556-019-0404-4PMC7354005

[137] KimH-S, KimHJ, LeeMR, HanI (2021). EMMPRIN expression is associated with metastatic progression in osteosarcoma. BMC cancer, 21:1059.34565336 10.1186/s12885-021-08774-9PMC8474954

[138] YoshidaK, TsudaM, MatsumotoR, SembaS, WangL, SuginoH, et al. (2019). Exosomes containing ErbB2/CRK induce vascular growth in premetastatic niches and promote metastasis of bladder cancer. Cancer Science, 110:2119-2132.31141251 10.1111/cas.14080PMC6609816

[139] XingS, HuK, WangY (2022). Tumor Immune Microenvironment and Immunotherapy in Non-Small Cell Lung Cancer: Update and New Challenges. Aging Dis, 13:1615-1632.36465180 10.14336/AD.2022.0407PMC9662266

[140] Gluszko A, Mirza SM, Piszczatowska K, Kantor I, Struga M, Szczepanski MJ (2019). The role of tumor-derived exosomes in tumor angiogenesis and tumor progression. Current Issues in Pharmacy and Medical Sciences, 32:193-202.

[141] MacklinA, KhanS, KislingerT (2020). Recent advances in mass spectrometry based clinical proteomics: applications to cancer research. Clinical Proteomics, 17:17.32489335 10.1186/s12014-020-09283-wPMC7247207

[142] Zhang H, Liu C, Wang S, Wang Q, Feng X, Jiang H, et al. (2024). Proteogenomic analysis of air-pollution-associated lung cancer reveals prevention and therapeutic opportunities. 2024.2003.2011.24304129. 10.7554/eLife.95453 PMC11493407 39432560

[143] BhawalR, ObergAL, ZhangS, KohliM (2020). Challenges and Opportunities in Clinical Applications of Blood-Based Proteomics in Cancer. Cancers, 12:2428.32867043 10.3390/cancers12092428PMC7564506

[144] MaoL, ChenJ, LuX, YangC, DingY, WangM, et al. (2021). Proteomic analysis of lung cancer cells reveals a critical role of BCAT1 in cancer cell metastasis. Theranostics, 11:9705-9720.34646394 10.7150/thno.61731PMC8490523

[145] DuanQ, LiD, XiongL, ChangZ, XuG (2019). SILAC Quantitative Proteomics and Biochemical Analyses Reveal a Novel Molecular Mechanism by Which ADAM12S Promotes the Proliferation, Migration, and Invasion of Small Cell Lung Cancer Cells through Upregulating Hexokinase 1. Journal of Proteome Research, 18:2903-2914.31117637 10.1021/acs.jproteome.9b00208

[146] Gasparri R, Noberini R, Cuomo A, Yadav A, Tricarico D, Salvetto C, et al. (2023). Serum proteomics profiling identifies a preliminary signature for the diagnosis of early-stage lung cancer. PROTEOMICS - Clinical Applications: 2200093. 10.1002/prca.202200093 36645712

[147] ZhangC, LengW, SunC, LuT, ChenZ, MenX, et al. (2018). Urine Proteome Profiling Predicts Lung Cancer from Control Cases and Other Tumors. EBioMedicine, 30:120-128.29576497 10.1016/j.ebiom.2018.03.009PMC5952250

[148] BöttgerF, Schaaij-VisserTB, de ReusI, PiersmaSR, PhamTV, NagelR, et al. (2019). Proteome analysis of non-small cell lung cancer cell line secretomes and patient sputum reveals biofluid biomarker candidates for cisplatin response prediction. Journal of Proteomics, 196:106-119.30710758 10.1016/j.jprot.2019.01.018

[149] LuJ, ZhangW, YuK, ZhangL, LouY, GuP, et al. (2022). Screening anlotinib responders via blood-based proteomics in non-small cell lung cancer. FASEB journal: official publication of the Federation of American Societies for Experimental Biology, 36:e22465.35867072 10.1096/fj.202101658R

[150] XuJ-Y, ZhangC, WangX, ZhaiL, MaY, MaoY, et al. (2020). Integrative Proteomic Characterization of Human Lung Adenocarcinoma. Cell, 182:245-261.e217.32649877 10.1016/j.cell.2020.05.043

[151] SuwinskiR, GiglokM, Galwas-KliberK, IdasiakA, JochymekB, DejaR, et al. (2019). Blood serum proteins as biomarkers for prediction of survival, locoregional control and distant metastasis rate in radiotherapy and radio-chemotherapy for non-small cell lung cancer. BMC cancer, 19:427.31068179 10.1186/s12885-019-5617-1PMC6507220

[152] PinhoSS, ReisCA (2015). Glycosylation in cancer: mechanisms and clinical implications. Nature Reviews Cancer, 15:540-555.26289314 10.1038/nrc3982

[153] MeanyDL, ChanDW (2011). Aberrant glycosylation associated with enzymes as cancer biomarkers. Clinical Proteomics, 8:7.21906357 10.1186/1559-0275-8-7PMC3170274

[154] KobataA, AmanoJ (2005). Altered glycosylation of proteins produced by malignant cells, and application for the diagnosis and immunotherapy of tumours. Immunology & Cell Biology, 83:429-439.16033539 10.1111/j.1440-1711.2005.01351.x

[155] DennisJ (1999). Glycoprotein glycosylation and cancer progression. Biochimica et Biophysica Acta (BBA) - General Subjects, 1473:21-34.10580127 10.1016/s0304-4165(99)00167-1

[156] VajariaBN, PatelPS (2017). Glycosylation: a hallmark of cancer? Glycoconjugate Journal, 34:147-156.27975160 10.1007/s10719-016-9755-2

[157] Dall'Olio F (2012). Mechanisms of cancer-associated glycosylation changes. Frontiers in Bioscience, 17:670. 10.2741/3951 22201768

[158] HiraoY, MatsuzakiH, IwakiJ, KunoA, KajiH, OhkuraT, et al. (2014). Glycoproteomics approach for identifying Glycobiomarker candidate molecules for tissue type classification of non-small cell lung carcinoma. Journal of Proteome Research, 13:4705-4716.25244057 10.1021/pr5006668

[159] KondoK, HaradaY, NakanoM, SuzukiT, FukushigeT, HanzawaK, et al. (2022). Identification of distinct N-glycosylation patterns on extracellular vesicles from small-cell and non-small-cell lung cancer cells. The Journal of Biological Chemistry, 298:101950.35447118 10.1016/j.jbc.2022.101950PMC9117544

[160] WenC-L, ChenK-Y, ChenC-T, ChuangJ-G, YangP-C, ChowL-P (2012). Development of an AlphaLISA assay to quantify serum core-fucosylated E-cadherin as a metastatic lung adenocarcinoma biomarker. Journal of Proteomics, 75:3963-3976.22634079 10.1016/j.jprot.2012.05.015

[161] JinY, YangY, SuY, YeX, LiuW, YangQ, et al. (2019). Identification a novel clinical biomarker in early diagnosis of human non-small cell lung cancer. Glycoconjugate Journal, 36:57-68.30607521 10.1007/s10719-018-09853-z

[162] LuH-H, LinS-Y, WengRR, JuanY-H, ChenY-W, HouH-H, et al. (2020). Fucosyltransferase 4 shapes oncogenic glycoproteome to drive metastasis of lung adenocarcinoma. EBioMedicine, 57:102846.32629386 10.1016/j.ebiom.2020.102846PMC7339020

[163] AlvarezMRS, ZhouQ, GrijaldoSJB, LebrillaCB, NacarioRC, HeraldeFM, et al. (2022). An Integrated Mass Spectrometry-Based Glycomics-Driven Glycoproteomics Analytical Platform to Functionally Characterize Glycosylation Inhibitors. Molecules (Basel, Switzerland), 27:3834.35744954 10.3390/molecules27123834PMC9228227

[164] ZengW, ZhengS, MaoY, WangS, ZhongY, CaoW, et al. (2021). Elevated N-Glycosylation Contributes to the Cisplatin Resistance of Non-Small Cell Lung Cancer Cells Revealed by Membrane Proteomic and Glycoproteomic Analysis. Frontiers in Pharmacology, 12:805499.35002739 10.3389/fphar.2021.805499PMC8728018

[165] AzmiAS, BaoB, SarkarFH (2013). Exosomes in cancer development, metastasis, and drug resistance: a comprehensive review. Cancer and Metastasis Reviews, 32:623-642.23709120 10.1007/s10555-013-9441-9PMC3843988

[166] CuiS, ChengZ, QinW, JiangL (2018). Exosomes as a liquid biopsy for lung cancer. Lung Cancer (Amsterdam, Netherlands), 116:46-54.29413050 10.1016/j.lungcan.2017.12.012

[167] SunY, LiuS, QiaoZ, ShangZ, XiaZ, NiuX, et al. (2017). Systematic comparison of exosomal proteomes from human saliva and serum for the detection of lung cancer. Analytica Chimica Acta, 982:84-95.28734369 10.1016/j.aca.2017.06.005

[168] HuangH, YangY, ZhuY, ChenH, YangY, ZhangL, et al. (2022). Blood protein biomarkers in lung cancer. Cancer Lett, 551:215886.35995139 10.1016/j.canlet.2022.215886

[169] Sandfeld-PaulsenB, JakobsenKR, BækR, FolkersenBH, RasmussenTR, MeldgaardP, et al. (2016). Exosomal Proteins as Diagnostic Biomarkers in Lung Cancer. Journal of Thoracic Oncology: Official Publication of the International Association for the Study of Lung Cancer, 11:1701-1710.27343445 10.1016/j.jtho.2016.05.034

[170] JeongH, ChoiBH, ParkJ, JungJ-H, ShinH, KangK-W, et al. (2021). GCC2 as a New Early Diagnostic Biomarker for Non-Small Cell Lung Cancer. Cancers, 13.34771645 10.3390/cancers13215482PMC8582534

[171] WangN, SongX, LiuL, NiuL, WangX, SongX, et al. (2018). Circulating exosomes contain protein biomarkers of metastatic non-small-cell lung cancer. Cancer Science, 109:1701-1709.29573061 10.1111/cas.13581PMC5980308

[172] GaoJ, QiuX, LiX, FanH, ZhangF, LvT, et al. (2018). Expression profiles and clinical value of plasma exosomal Tim-3 and Galectin-9 in non-small cell lung cancer. Biochemical and Biophysical Research Communications, 498:409-415.29452091 10.1016/j.bbrc.2018.02.114

[173] LiuS, TianW, MaY, LiJ, YangJ, LiB (2022). Serum exosomal proteomics analysis of lung adenocarcinoma to discover new tumor markers. BMC cancer, 22:279.35291954 10.1186/s12885-022-09366-xPMC8925168

[174] PedersenS, JensenKP, HonoreB, KristensenSR, PedersenCH, SzejniukWM, et al. (2022). Circulating microvesicles and exosomes in small cell lung cancer by quantitative proteomics. Clin Proteomics, 19:2.34996345 10.1186/s12014-021-09339-5PMC8903681

[175] SunY, HuoC, QiaoZ, ShangZ, UzzamanA, LiuS, et al. (2018). Comparative Proteomic Analysis of Exosomes and Microvesicles in Human Saliva for Lung Cancer. Journal of proteome research, 17:1101-1107.29397740 10.1021/acs.jproteome.7b00770

[176] LiY, ZhangY, QiuF, QiuZ (2011). Proteomic identification of exosomal LRG1: a potential urinary biomarker for detecting NSCLC. Electrophoresis, 32:1976-1983.21557262 10.1002/elps.201000598

[177] JinS, LiuT, WangW, LiT, LiuZ, ZhangM (2023). Lymphocyte migration regulation related proteins in urine exosomes may serve as a potential biomarker for lung cancer diagnosis. BMC Cancer, 23:1125.37980468 10.1186/s12885-023-11567-xPMC10656923

[178] LuoB, QueZ, LuX, QiD, QiaoZ, YangY, et al. (2023). Identification of exosome protein panels as predictive biomarkers for non-small cell lung cancer. Biol Proced Online, 25:29.37953280 10.1186/s12575-023-00223-0PMC10641949

[179] BaranK, WaśkoJ, KryczkaJ, BoncelaJ, JabłońskiS, KolesińskaB, et al. (2023). The Comparison of Serum Exosome Protein Profile in Diagnosis of NSCLC Patients. Int J Mol Sci, 24.37761972 10.3390/ijms241813669PMC10650331

[180] GagliardiF, NarayananA, MortiniP (2017). SPARCL1 a novel player in cancer biology. Critical Reviews in Oncology/Hematology, 109:63-68.28010899 10.1016/j.critrevonc.2016.11.013

[181] FengX, LiuX, XiangJ, XuJ, YinN, WangL, et al. (2023). Exosomal ITGB6 from dormant lung adenocarcinoma cells activates cancer-associated fibroblasts by KLF10 positive feedback loop and the TGF-β pathway. Transl Lung Cancer Res, 12:2520-2537.38205211 10.21037/tlcr-23-707PMC10775012

[182] HanR, GuoH, ShiJ, WangH, ZhaoS, JiaY, et al. (2023). Tumour microenvironment changes after osimertinib treatment resistance in non-small cell lung cancer. Eur J Cancer, 189:112919.37320935 10.1016/j.ejca.2023.05.007

[183] YangH, SunB, FanL, MaW, XuK, HallSRR, et al. (2022). Multi-scale integrative analyses identify THBS2(+) cancer-associated fibroblasts as a key orchestrator promoting aggressiveness in early-stage lung adenocarcinoma. Theranostics, 12:3104-3130.35547750 10.7150/thno.69590PMC9065207

[184] WelshJA, GoberdhanDCI, O'DriscollL, BuzasEI, BlenkironC, BussolatiB, et al. (2024). Minimal information for studies of extracellular vesicles (MISEV2023): From basic to advanced approaches. J Extracell Vesicles, 13:e12404.38326288 10.1002/jev2.12404PMC10850029

[185] Van DeunJ, HendrixA (2017). Is your article EV-TRACKed? J Extracell Vesicles, 6:1379835.29184624 10.1080/20013078.2017.1379835PMC5698936

[186] PathanM, FonsekaP, ChittiSV, KangT, SanwlaniR, Van DeunJ, et al. (2019). Vesiclepedia 2019: a compendium of RNA, proteins, lipids and metabolites in extracellular vesicles. Nucleic Acids Res, 47:D516-d519.30395310 10.1093/nar/gky1029PMC6323905

[187] KeerthikumarS, ChisangaD, AriyaratneD, Al SaffarH, AnandS, ZhaoK, et al. (2016). ExoCarta: A Web-Based Compendium of Exosomal Cargo. J Mol Biol, 428:688-692.26434508 10.1016/j.jmb.2015.09.019PMC4783248

[188] Feng J, Ding C, Qiu N, Ni X, Zhan D, Liu W, et al. (2017). Firmiana: towards a one-stop proteomic cloud platform for data processing and analysis. Nature Biotechnology, 35:409-412. 10.1038/nbt.3825 28486446

[189] TaylorCF, PatonNW, LilleyKS, BinzPA, JulianRKJr., JonesAR, et al. (2007). The minimum information about a proteomics experiment (MIAPE). Nat Biotechnol, 25:887-893.17687369 10.1038/nbt1329

[190] DeutschEW, LaneL, OverallCM, BandeiraN, BakerMS, PineauC, et al. (2019). Human Proteome Project Mass Spectrometry Data Interpretation Guidelines 3.0. J Proteome Res, 18:4108-4116.31599596 10.1021/acs.jproteome.9b00542PMC6986310

[191] Dumas T, Martinez Pinna R, Lozano C, Radau S, Pible O, Grenga L, et al.2024. The astounding exhaustiveness and speed of the Astral mass analyzer for highly complex samples is a quantum leap in the functional analysis of microbiomes. England. 10.1186/s40168-024-01766-4 PMC10918999 38454512

[192] Guzman UH, Martinez-Val A, Ye Z, Damoc E, Arrey TN, Pashkova A, et al. (2024). Ultra-fast label-free quantification and comprehensive proteome coverage with narrow-window data-independent acquisition. Nature biotechnology. 10.1038/s41587-023-02099-7 PMC11631760 38302753

[193] Lancaster NM, Sinitcyn P, Forny P, Peters-Clarke TM, Fecher C, Smith AJ, et al. (2023). Fast and Deep Phosphoproteome Analysis with the Orbitrap Astral Mass Spectrometer. 10.1038/s41467-024-51274-0 PMC11327265 39147754

[194] P B, I P, C K, C G, A.m dV, J.v O (2023). Systematic optimization of automated phosphopeptide enrichment for high-sensitivity phosphoproteomics. bioRxiv. 10.1016/j.mcpro.2024.100754 PMC11087715 38548019

[195] StewartHI, GrinfeldD, GiannakopulosA, PetzoldtJ, ShanleyT, GarlandM, et al. (2023). Parallelized Acquisition of Orbitrap and Astral Analyzers Enables High-Throughput Quantitative Analysis. Analytical chemistry, 95:15656-15664.37815927 10.1021/acs.analchem.3c02856PMC10603608

[196] HeilLR, DamocE, ArreyTN, PashkovaA, DenisovE, PetzoldtJ, et al. (2023). Evaluating the Performance of the Astral Mass Analyzer for Quantitative Proteomics Using Data-Independent Acquisition. Journal of proteome research, 22:3290-3300.37683181 10.1021/acs.jproteome.3c00357PMC10563156

[197] V P, P A-F, T.n A, N U, B F, H S, et al. (2023). Evaluating the capabilities of the Astral mass analyzer for single-cell proteomics. bioRxiv.

[198] U.h G, A.m DV, Z Y, E D, T.n A, A P, et al. (2023). Narrow-window DIA: Ultra-fast quantitative analysis of comprehensive proteomes with high sequencing depth. bioRxiv.

[199] ZhaoY, XueQ, WangM, MengB, JiangY, ZhaiR, et al. (2023). Evolution of Mass Spectrometry Instruments and Techniques for Blood Proteomics. J Proteome Res, 22:1009-1023.36932955 10.1021/acs.jproteome.3c00102

[200] PetreraA, von ToerneC, BehlerJ, HuthC, ThorandB, HilgendorffA, et al. (2021). Multiplatform Approach for Plasma Proteomics: Complementarity of Olink Proximity Extension Assay Technology to Mass Spectrometry-Based Protein Profiling. J Proteome Res, 20:751-762.33253581 10.1021/acs.jproteome.0c00641

[201] CanoA, Esteban-de-AntonioE, BernuzM, PuertaR, García-GonzálezP, de RojasI, et al. (2023). Plasma extracellular vesicles reveal early molecular differences in amyloid positive patients with early-onset mild cognitive impairment. J Nanobiotechnology, 21:54.36788617 10.1186/s12951-023-01793-7PMC9930227

[202] DingL, LiX, ZhuH, LuoH (2022). Single-Cell Sequencing in Rheumatic Diseases: New Insights from the Perspective of the Cell Type. Aging Dis, 13:1633-1651.36465169 10.14336/AD.2022.0323PMC9662270

[203] GrossA, SchoendubeJ, ZimmermannS, SteebM, ZengerleR, KoltayP (2015). Technologies for Single-Cell Isolation. Int J Mol Sci, 16:16897-16919.26213926 10.3390/ijms160816897PMC4581176

[204] LohaniV, A RA, KunduS, AkhterMQ, BagS (2023). Single-Cell Proteomics with Spatial Attributes: Tools and Techniques. ACS omega, 8:17499-17510.37251119 10.1021/acsomega.3c00795PMC10210017

[205] BudnikB, LevyE, HarmangeG, SlavovN (2018). SCoPE-MS: mass spectrometry of single mammalian cells quantifies proteome heterogeneity during cell differentiation. Genome Biol, 19:161.30343672 10.1186/s13059-018-1547-5PMC6196420

[206] SpitzerMH, NolanGP (2016). Mass Cytometry: Single Cells, Many Features. Cell, 165:780-791.27153492 10.1016/j.cell.2016.04.019PMC4860251

[207] MooreJL, CharkoftakiG (2023). A Guide to MALDI Imaging Mass Spectrometry for Tissues. J Proteome Res, 22:3401-3417.37877579 10.1021/acs.jproteome.3c00167

[208] ItzhakDN, DaviesC, TyanovaS, MishraA, WilliamsonJ, AntrobusR, et al. (2017). A Mass Spectrometry-Based Approach for Mapping Protein Subcellular Localization Reveals the Spatial Proteome of Mouse Primary Neurons. Cell Rep, 20:2706-2718.28903049 10.1016/j.celrep.2017.08.063PMC5775508

[209] KimDI, RouxKJ (2016). Filling the Void: Proximity-Based Labeling of Proteins in Living Cells. Trends Cell Biol, 26:804-817.27667171 10.1016/j.tcb.2016.09.004PMC5077660

[210] TorresNP, HoB, BrownGW (2016). High-throughput fluorescence microscopic analysis of protein abundance and localization in budding yeast. Crit Rev Biochem Mol Biol, 51:110-119.26893079 10.3109/10409238.2016.1145185

[211] SorinM, RezanejadM, KarimiE, FisetB, DesharnaisL, PerusLJM, et al. (2023). Single-cell spatial landscapes of the lung tumour immune microenvironment. Nature, 614:548-554.36725934 10.1038/s41586-022-05672-3PMC9931585

[212] KarimiE, YuMW, MaritanSM, PerusLJM, RezanejadM, SorinM, et al. (2023). Single-cell spatial immune landscapes of primary and metastatic brain tumours. Nature, 614:555-563.36725935 10.1038/s41586-022-05680-3PMC9931580

[213] ArslanT, PanY, MermelekasG, VesterlundM, OrreLM, LehtiöJ (2022). SubCellBarCode: integrated workflow for robust spatial proteomics by mass spectrometry. Nat Protoc, 17:1832-1867.35732783 10.1038/s41596-022-00699-2

[214] Luecken MD, Zaragosi L-E, Madissoon E, Sikkema L, Firsova AB, De Domenico E, et al. (2022). The discovAIR project: a roadmap towards the Human Lung Cell Atlas. The European respiratory journal, 60. 10.1183/13993003.02057-2021 PMC9386332 35086829

[215] LinD, ChenX, LiuY, LinZ, LuoY, FuM, et al. (2021). Microgel Single-Cell Culture Arrays on a Microfluidic Chip for Selective Expansion and Recovery of Colorectal Cancer Stem Cells. Anal Chem, 93:12628-12638.34495647 10.1021/acs.analchem.1c02335

[216] MasicaDL, KarchinR (2011). Correlation of somatic mutation and expression identifies genes important in human glioblastoma progression and survival. Cancer Res, 71:4550-4561.21555372 10.1158/0008-5472.CAN-11-0180PMC3129415

[217] YangM, PetraliaF, LiZ, LiH, MaW, SongX, et al. (2020). Community Assessment of the Predictability of Cancer Protein and Phosphoprotein Levels from Genomics and Transcriptomics. Cell Syst, 11:186-195.e189.32710834 10.1016/j.cels.2020.06.013

[218] PayneSH (2015). The utility of protein and mRNA correlation. Trends Biochem Sci, 40:1-3.25467744 10.1016/j.tibs.2014.10.010PMC4776753

[219] ArshadOA, DannaV, PetyukVA, PiehowskiPD, LiuT, RodlandKD, et al. (2019). An Integrative Analysis of Tumor Proteomic and Phosphoproteomic Profiles to Examine the Relationships Between Kinase Activity and Phosphorylation. Mol Cell Proteomics, 18:S26-s36.31227600 10.1074/mcp.RA119.001540PMC6692771

[220] JoshiSK, PiehowskiP, LiuT, GoslineSJC, McDermottJE, DrukerBJ, et al. (2024). Mass Spectrometry-Based Proteogenomics: New Therapeutic Opportunities for Precision Medicine. Annu Rev Pharmacol Toxicol, 64:455-479.37738504 10.1146/annurev-pharmtox-022723-113921PMC10950354

[221] LuoHT, ZhengYY, TangJ, ShaoLJ, MaoYH, YangW, et al. (2021). Dissecting the multi-omics atlas of the exosomes released by human lung adenocarcinoma stem-like cells. NPJ Genom Med, 6:48.34127680 10.1038/s41525-021-00217-5PMC8203745

[222] HermannJ, SchurgersL, JankowskiV (2022). Identification and characterization of post-translational modifications: Clinical implications. Mol Aspects Med, 86:101066.35033366 10.1016/j.mam.2022.101066

[223] ZhaoL, WangH, FuJ, WuX, LiangXY, LiuXY, et al. (2022). Microfluidic-based exosome isolation and highly sensitive aptamer exosome membrane protein detection for lung cancer diagnosis. Biosens Bioelectron, 214:114487.35780540 10.1016/j.bios.2022.114487

[224] TongZ, YangD, ShenC, LiC, XuX, LiQ, et al. (2024). Rapid automated extracellular vesicle isolation and miRNA preparation on a cost-effective digital microfluidic platform. Anal Chim Acta, 1296:342337.38401929 10.1016/j.aca.2024.342337

[225] SinaAAI, VaidyanathanR, DeyS, CarrascosaLG, ShiddikyMJA, TrauM (2016). Real time and label free profiling of clinically relevant exosomes. Scientific Reports, 6:30460.27464736 10.1038/srep30460PMC4964344

[226] BoriachekK, MasudMK, PalmaC, PhanH-P, YamauchiY, HossainMSA, et al. (2019). Avoiding Pre-Isolation Step in Exosome Analysis: Direct Isolation and Sensitive Detection of Exosomes Using Gold-Loaded Nanoporous Ferric Oxide Nanozymes. Analytical Chemistry, 91:3827-3834.30735354 10.1021/acs.analchem.8b03619

[227] RahimianS, NajafiH, AfzaliB, DoroudianM (2024). Extracellular Vesicles and Exosomes: Novel Insights and Perspectives on Lung Cancer from Early Detection to Targeted Treatment. Biomedicines, 12.38255228 10.3390/biomedicines12010123PMC10813125

[228] McKiernanJ, DonovanMJ, MargolisE, PartinA, CarterB, BrownG, et al. (2018). A Prospective Adaptive Utility Trial to Validate Performance of a Novel Urine Exosome Gene Expression Assay to Predict High-grade Prostate Cancer in Patients with Prostate-specific Antigen 2-10ng/ml at Initial Biopsy. Eur Urol, 74:731-738.30237023 10.1016/j.eururo.2018.08.019

